# Discovery of Ethyl 2-Nitro-3-Arylacrylates Molecules as T3SS Inhibitor Reducing the Virulence of Plant Pathogenic Bacteria *Xanthomonas*

**DOI:** 10.3389/fmicb.2019.01874

**Published:** 2019-08-20

**Authors:** Shan Jiang, Hui Li, Wasim Ahmed, Xuwen Xiang, Gaopeng Song, Zi-Ning Cui

**Affiliations:** ^1^State Key Laboratory for Conservation and Utilization of Subtropical Agro-Bioresources, Integrative Microbiology Research Centre, Guangdong Province Key Laboratory of Microbial Signals and Disease Control, South China Agricultural University, Guangzhou, China; ^2^College of Materials and Energy, South China Agricultural University, Guangzhou, China

**Keywords:** *Xanthomonas oryzae* pv. *oryzae* (*Xoo*), *Xanthomonas campestris* pv. *campestris* (*Xcc*), type III secretion system (T3SS), anti-virulence compounds, ethyl 2-nitro-3-arylacrylates molecules

## Abstract

*Xanthomonas oryzae* pv. *oryzae* (*Xoo*) is a gram-negative pathogen which causes leaf blight disease. Known traditional bactericides are not much more effective in inhibiting this bacteria than before. Selecting the virulence factor of the bacteria as the target without affecting their growth has been considered as a novel method for developing new anti-microbial drugs. Type III secretion systems (T3SS) are one of the important and highly conserved virulence factors in most gram-negative pathogens, which has been considered as an effective target to develop new anti-microbial drugs. In order to discover potential anti-microbial drugs against *Xoo* pathogens, a series of ethyl 2-nitro-3-arylacrylates compounds were screened. Among them, the compounds I-9, I-12, and I-13 could highly inhibit the promoter activity of a harpin gene *hpa1*, which were used to further check for the influence on bacterial growth and on the hypersensitive response (HR) caused by *Xoo* bacteria on non-host plants. The results showed that above compounds could reduce HR without affecting bacterial growth and survival. Moreover, qRT-PCR analysis indicated that treatment with the three inhibitors (I-9, I-12, and I-13) could suppress the expression of the *Xoo* T3SS in different extent. The mRNA levels of representative genes in the *hrp* cluster, including the key regulatory genes *hrpG* and *hrpX*, were decreased. Last but not least, *in vivo* test ensured that the above compounds reduced the disease symptoms of *Xoo* on the rice and *Xcc* on the Chinese radish.

## Graphical Abstract

Small molecule inhibitors targeting type III secretion system of *Xanthomonas*.

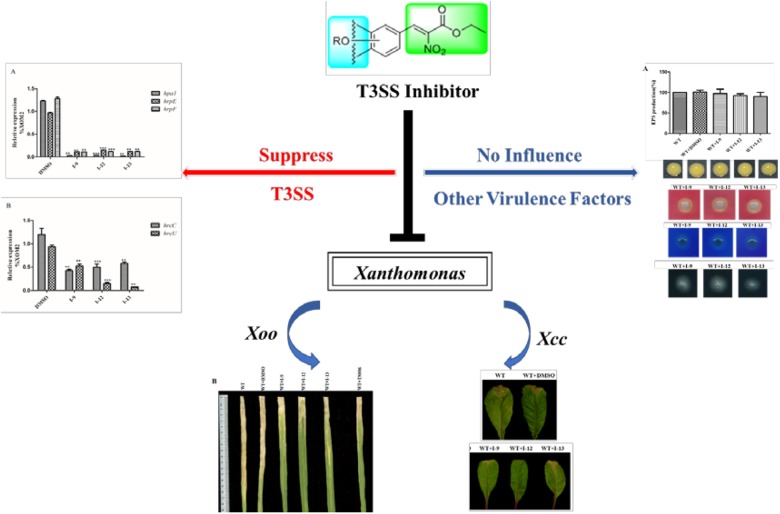

## Introduction

*Xanthomonas oryzae* pv. *oryzae* (*Xoo*) is a gram-negative rod-shaped bacterium which is an important source of bacterial disease on rice (Mansfield et al., 2012). It can cause the most serious bacterial leaf blight disease, with an annual yield loss of 10–50%, and even at 100% under severe conditions in some countries (Zhang and Wang, 2013; Sahu et al., 2018). In the past, traditional bactericides such as bismerthiazol and streptomycinc were mainly adopted to control this rice bacterial disease (Xu et al., 2010). However, resistance of *Xoo* against these bactericides has been observed recently (Shi et al., 2015; Yu et al., 2016), which generally affects the essential factors in the process of bacterial growth and survival (Rasko and Sperandio, 2010; Fan et al., 2017). Therefore, there is a demand for the development of novel classes of agents to combat resistance of *Xoo* that target bacterial virulence factors rather than their key processes of growth and survival (Barczak and Hung, 2009; Rasko and Sperandio, 2010).

A variety of virulence factors have been reported in *Xoo*, such as type III secretion system, exopolysaccharide (EPS), and type II secretion system (T2SS) that is used to secrete extracellular enzymes (Rajeshwari et al., 2005; Das et al., 2009; Rai et al., 2012). The type III secretion system (T3SS), as the key virulence factor in plant pathogenesis, is used to transport effector proteins into plants. T3SS is highly conserved among most gram-negative pathogenic bacteria and not necessary for bacterial growth and survival (Buttner, 2012; Xiang et al., 2018). Hence, it is considered to be an ideal target for the discovery and development of novel antimicrobial drugs (Marshall and Finlay, 2014; Charro and Mota, 2015). To date, various types of small molecules has been identified as T3SS inhibitors among different pathogens, including *Pseudomonas aeruginosa, Yersinia, Salmonella*, and *Dickeya dadantii* (Nordfelth et al., 2005; Hudson et al., 2007; Li et al., 2009; Yamazaki et al., 2012), which can reduce effectively the likelihood of side effects (Felise et al., 2008). It was proved that these inhibitors may directly affect the components of the T3SS apparatus (Bowlin et al., 2014; Jessen et al., 2014), or the function of regulating T3SS gene expression (Guo et al., 2005; Yang et al., 2014), or through several indirect interactions (Petnicki-Ocwieja et al., 2005).

Like many gram-negative bacteria, *Xoo* delivers effector proteins into host plant cells by the T3SS apparatus, which is encoded by the gene locus of hypersensitive response and pathogenicity (*hrp*) with more than 20 genes (Cho et al., 2008). The core operon includes *hrp*, *hrc* (*hrp*-conserved) and *hpa* (*hrp*-associated) genes (Zou et al., 2006). The T3SS apparatus confers the pathogenicity of bacteria on host plants and touches off hypersensitive response (HR) on non-host or resistant plants (Alfano and Collmer, 1997). *Hrp* gene expression is induced in planta and suppressed in nutrient-rich medium (Tang et al., 2006). *Xanthomonas* spp. and *Ralstonia solanacearum* are classified as *hrp* group II, which are not the same as group I in *P. syringae* and *Erwinia amylovora* (Van et al., 1995; Tang et al., 2006). In group II, many *hrp* genes have a similar sequence called Plant Inducible Promoter (PIP) box in their promoter region and their expression is regulated by *hrpG* and *hrpX*, which are located spatially away from the *hrp* gene cluster (Rasko and Sperandio, 2010). HrpG belongs to the OmpR family of two-component signal transduction systems (TCS), which is able to regulate the expression of *hrpX* positively (Wengelnik et al., 1996). HrpX belongs to the AraC family, which activates other *hrp* genes’ transcription (*hrpB* to *hrpF*), together with the genes encoding T3SS effectors (Buttner et al., 2004). In the HrpG regulon, HrpX regulates most genes that are have an interaction with PIP-box and many T3SS effectors have PIP-box in their promoter region.

**FIGURE 1:**
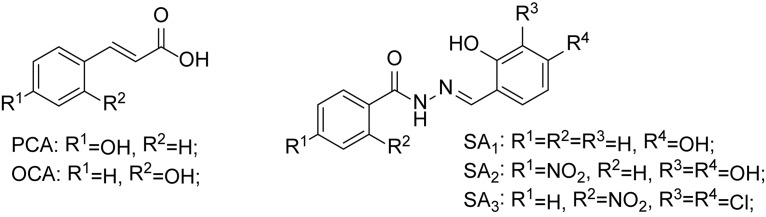
The chemical structures of PCA, OCA, and SA_1__–__3_.

**FIGURE 2:**
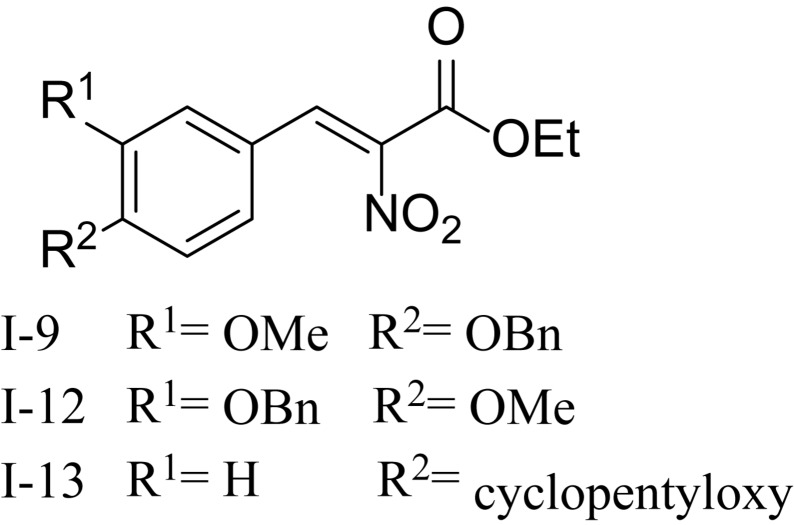
The chemical structures of compounds I-9, I-12, and I-13.

Recently, it has been discovered that some phenolic compounds such as *p*-coumaric acid (PCA) and their derivatives (Figure 1) are able to suppress T3SS functionality in *X. oryzae* (Fan et al., 2017), *D. dadantii* (Li et al., 2009), and *E. amylovora* (Yang et al., 2007), respectively. Particularly, TS006 (*o*-coumaric acid, OCA), as a member of phenylpropanoic acids as do PCA, revealed the potential effect in suppressing the disease symptoms caused by *Xoo* and *Xoc* on rice leaves (Fan et al., 2017). Recent reports have shown that salicylidene acylhydrazides SA_1__–__3_ proved to be a powerful inhibitor against the *R. solanacearum* T3SS (Figure 1) (Puigvert et al., 2019). Structurally, salicylidene acylhydrazides were derived from OCA on the basis of hybridization principle and bioisosterism, suggesting that both modification of the COOH group and introduction of some suitable groups containing N such as NO_2_ in the phenylpropanoic acids could be helpful to obtain the new T3SS inhibitors. Based on the above observations, we have synthesized and screened a series of ethyl 2-nitro-3-arylacrylates I-1 to I-24 (Supplementary Figure S1) and found three of them, I-9, I-12 and I-13 (Figure 2) exhibited stronger inhibition against the T3SS expression of *Xoo* compared to the positive reference compound TS006. The compounds I-9, I-12, and I-13 were selected for further analysis, which suppressed HR on tobacco without killing bacteria. The mechanism of the above three inhibitors was demonstrated by testing the effects on expression of typical *hrp* genes by qRT-PCR. Compounds I-9, I-12, and I-13 do not affect other typical virulence factors and motility in *Xoo*, such as EPS, extracellular cellulase, and extracellular xylanase. Pathogenicity assays indicated that these inhibitors weaken the disease symptoms caused by *Xoo* and *Xcc*. The *in vivo* results demonstrated that the compounds showed better protective activity against rice bacterial leaf blight than commercial antibacterial drugs thiodiazole copper and bismerthiazol in greenhouse test.

## Materials and Methods

### Instrumental Analysis

Solvents were purified in a conventional manner. Thin layer chromatography (TLC) was performed on precoated E. Merck silica gel 60 F254 plates. Flash column chromatography was performed on silica gel (200-300 mesh, Qingdao, China). ^1^H NMR and ^13^C NMR spectra were measured on Bruker DPX400 and Bruker AV600 (Bruker, Fallanden, Switzerland), while tetramethylsilane as an internal standard, and chemical shifts were recorded in ppm values. The promoter activity of *hpa1* was checked by a FACS-Caliber flow cytometer (CytoFLEX, United States). The growth rates were recorded using a Bioscreen (Bioscreen, Finland). RNA concentration and purity were monitored using the Nanovue UV-Vs spectrophotometer (GE Healthcare Bio-Science, Sweden). The cDNA levels were quantified by real-time PCR using a SYBR Green Master Mix (Thermo, United States).

### General Procedure

#### Synthesis of Title Compounds (I-1 to I-24)

All the compounds were synthesized following our previous literatures (Fan et al., 2017; Song et al., 2017). The procedures and identifications were listed in the Supplementary Materials.

### Bioassays

#### Bacterial Strains, Plasmids and Growth Conditions

The plasmids, bacterial strains and primers used in this experimental are listed in Supplementary Table S1. PXO99^*A*^ strain (*Xoo* wild-type strain) and the derived strains were grown in M210 medium which were prepared as reported or on PSA plates for growth studies (Tsuge et al., 2002). *Escherichia coli* was grown in Luria Bertani (LB) medium at 37°C. XOM2 medium were prepared as reported (Tsuge et al., 2002).

In *Xanthomonas campestris pv. Campestris 8004 (Xcc8004*), a 300 bp fragment carrying the promoter region of *hpa1*, was PCR amplified by using Phpa1-F and Phpa1-R primers in Supplementary Figure S1. The fragment was linked to pPROBE-AT, which was a broad-host-range vector containing a promoter-less GFP gene.

Ampicillin (Ap) and cephalexin (Cp) were used at the final concentrations of 10 μg/mL and 25 μg/mL.

All the compounds were dissolved in dimethyl sulfoxide (DMSO) and DMSO was used as a solvent control.

### Flow Cytometry Analysis

*Xoo* PXO99^*A*^ including the pPhpa1 and promoterless pPROBE-AT was cultured in M210 overnight at 28°C, then transferred to XOM2 added with 100 μM of each compound and shaking 15 h and suspended in PBS (PH = 7.4). The promoter activity of *hpa1* was tested by a FACS-Caliber flow cytometer.

Similar methods were applied to analyze the promoter activities of *hrpG*, *hrpX*, *hrcT* and pPhpa1 in *Xcc* 8004 which was grown in NYGB medium at 28°C overnight and then transferred to XCM2 medium (Slater et al., 2000).

The same volume of DMSO was used as a control. Three independent experiments were performed, and three replicates experiments were done each time.

### Measurement of the Growth Rate

*Xoo* cells were cultured overnight at 28°C in M210. The *Xoo* cells suspensions were adjusted to OD_600_ ≈ 1.0, then transferred into M210 or XOM2 (plus 0.5% sucrose) medium containing 100 μM of tested compounds or DMSO, starting at an OD_600_ of 0.1. The growth rates were recorded every 2 h during the 48 h period using a Bioscreen. Three independent experiments were performed, and three replicates were performed in each experiment.

### HR Assay

*Xoo* cells were grown in M210 to OD_600_ ≈ 2.0 at 28°C, and then suspended in sterile distilled water. The *Xoo* cells suspensions were adjusted to OD_600_ ≈ 0.5. Before infiltration into tobacco using a 1.0 mL needleless syringe, the cells suspensions were incubated at 28°C for 2 h with 100 μM of tested compounds or DMSO.

The similar methods were applied to do HR assay in *Xcc* cells.

All the HR symptoms appeared at 24 h after inoculation, then the symptoms were recorded by photographing. And *Nicotiana benthamiana* plants were used for all HR assays.

### RNA Extraction, cDNA Synthesis and qRT-PCR Analysis

*Xoo* cells were grown in M210 overnight at 28°C and subcultured to XOM2 at OD_600_ ≈ 0.6, added into DMSO or 100 μM of tested compounds and grew at 28°C for 15 h. Total RNA was extracted by using an RNAprep Pure Bacteria Kit (Promega, United States). cDNA was synthesized using an HiScriptII Q RT SuperMix Kit (Tiangen, Beijing, China). The relative expression levels of mRNA in the samples were quantified by real-time PCR using a SYBR Green Master Mix. The relative expression levels of mRNA were calculated and analyzed by the 2^–ΔΔ*CT*^ method (Livak and Schmittgen, 2001). The DNA gyrase subunit B (*gyrB*) gene was used as the internal control (Tsuge et al., 2002).

### Pathogenicity Assays

*Xoo* cells were grown in M210 to OD_600_ ≈ 2.0 at 28°C, and suspended in sterile distilled water. The *Xoo* cells suspensions were adjusted to OD_600_ ≈ 0.8 and treated with 100 μM of the tested compounds or DMSO, then incubated for 2 h at 28°C. The rice cultivar IR24 (*Oryza sativa* ssp. *indica*) was used for this experiment. *Xoo* bacterial cells were respectively inoculated on seedlings (2-week-old, by using a 1.0 mL needleless syringe) and adult plants (2-month-old, by the leaf snipping inoculation method). The symptoms of plants were scored and recorded by photographing at 3 days post-inoculation in seedlings, and at 14 days post-infection for lesion lengths in adult rice plants.

Chinese radish was used in the experiments, which grew to the 4-leaf stage. *Xcc* cells were grown in NYGB to OD_600_ ≈ 2.0 at 28°C and suspended in sterile distilled water. The *Xcc* cells suspensions were adjusted to OD_600_ ≈ 0.8 and treated with 100 μM of the tested compounds or DMSO, then incubated for 2 h at 28°C. The next step was to use sterile surgical scissors to delay post-infection the bacterial solution, and inoculated the leaves perpendicular to the midrib which was about 0.5 cm from the tip of the leaf. During the experiments, plants were cultured in a greenhouse at 25°C (16 h of light and 8 h of darkness).

### Motility Assay

*Xoo* cells were grown in M210 at 28°C for 24 h, and suspended in sterile distilled water to 1 × 10^9^ cells/mL. Then, 2 μL of these cell suspensions were inoculated onto the surface of plates containing 0.03% Bacto peptone, 0.03% yeast extract, and 0.3% agar, and maintained at 28°C for 3 days (Shen et al., 2001). The experimental phenomena were scrutinized after bacterial growth for 3 days. The swimming motility was compared by measuring the sizes of the zone between wild-type and wild-type containing compounds. The experiment was repeated three times.

### EPS and Extracellular Hydrolytic Enzymes Production Assay

For analyzing EPS production, the supernatants of bacterial culture (100 ml, OD_600_ = 2.0) were collected by centrifugation at 12,000 rpm for 10 min. To the supernatants were added two volumes of absolute ethanol and kept at −20°C for at least 15 h. The mixtures were centrifuged, and the precipitates of EPS were dried at 50°C overnight before determination of dry weight (Yang et al., 2012).

For analyzing production of extracellular cellulase activity and extracellular xylanase activity, the following steps were completed as described (Chatterjee et al., 1995; Furutani et al., 2003). PSA plates containing 0.5% carboxymethyl cellulose were used to analyze extracellular cellulase activity. The plates were dyed with 0.1% Congo red for 20 min and washed twice with 1.0 M NaCl. Cellulase-positive colonies could show pale-yellow clear zones against a red background. Xylanase activity was tested on the PSA plate which contained 0.2% RBB-xylan by the appearance of white clear zones among a blue background. The experiments were repeated three times.

### *In vivo* Protection Activity Test

The protection activity of title compounds against rice bacterial leaf blight in potted plants was conducted under greenhouse conditions. The procedures were followed by the literatures (Li et al., 2018).

### Statistical Analysis

GraphPad Prism 6.0 software was used to perform statistical analyses. Results were analyzed by the Student’s *t*-test (two-tailed).

## Results and Discussion

### Effect of the Screening Compounds on T3SS of *Xoo*

In order to make sure that the compounds could affect the expression of T3SS in *Xoo*, a library of compounds were used to test their effects on the promoter activity of the *hpa1* gene (Table 1), whose expression is induced in the hrp-inducing medium XOM2. *Hpa1* encodes a harpin protein in *Xoo* which could trigger HR in tobacco, and the HrpX regulated the *hpa1* gene expression. Wild-type strain having pPhpa1 was added to the tested compounds at 100 μM, and then cultured in XOM2 for 15 h before measuring the promoter activity of *hpa1*. All the compounds were tested for alterations in *hpa1* promoter activity by utilizing highly efficient fluorescence activated cell sorting (FACS) system. The mean fluorescence intensity (MFI) was listed in Table 1, meaning the promoter activity of *hpa1*. The ratio of MFI after adding the compounds to that of the DMSO control was calculated, and the results were listed in Table 1 indicated by %DMSO. The calculation method was the ratio of the MFI of the solvent after treatment of each compound to the MFI of the DMSO solvent control.

**TABLE 1:** Screening for inhibitors of *Xoo* T3SS by fluorescence-activated cell sorting assays.

**Compound**	**Avg MFI ± SD^a^**	**DMSO%^b^**	**Inhibition rate% (100% – DMSO%)**
DMSO	2343.23442.81		
I-1	1460.93236.87	62.35	37.65
I-2	559.3786.50	23.87	76.13
I-3	798.0062.30	34.06	65.94
I-4	1217.77133.46	51.97	48.03
I-5	984.3020.70	42.01	57.99
I-6	597.1387.23	25.48	74.52
I-7	400.1345.16	17.08	82.92
I-8	236.107.90	10.08	89.92
I-9	30.97 + 13.51	0.30	99.02
I-11	614.8347.40	26.24	73.76
I-12	22.833.43	0.97	99.03
I-13	19.635.15	0.84	99.16
I-14	324.801.27	13.86	86.14
I-15	258.5330.89	11.03	88.97
I-16	244.303.70	10.42	89.58
I-17	283.2020.19	12.09	87.91
I-18	323.900.59	13.82	86.18
I-19	271.771.43	11.60	88.40
I-20	259.7365.09	11.08	88.92
I-21	1721.9073.50	73.48	26.52
I-22	1350.6555.75	57.64	42.36
I-23	246.2323.80	10.51	89.49
I-24	242.5017.35	10.35	89.65
TS006	87.8318.62	8.53 96.25	90.29

*^*a*^Green fluorescent protein (GFP) and mean fluorescence intensity (MFI) were measured for gated populations of bacterial cells by flow cytometry (bacterial cells cultured in XOM2 with DMSO or XOM2 added with 100 μM of each compound). Values are representative of at least three independent experiments, and three replicates were done for each experiment. ^*b*^The calculated method was following the formula: DMSO% = 100 × MFI (XOM2 with tested compounds)/MFI (XOM2 with DMSO). TS006 was used as the control. I-10 compound added to XOM2 medium which produced significant precipitation and could not be used for subsequent FACS detection.*

As shown in Table 1, 11 of the title compounds showed strong inhibition against the *hpa1* promoter activity over 80% at 100 μM. Among them, compounds I-9, I-12, I-13 exhibited the foremost inhibition activity, which exerted higher potential than the reference compound TS006. The primary structure-activity relationship revealed that incorporation of the rigid alkoxy substituents only at the *meta*- or *para*- benzene position was helpful to enhance the inhibitory activity, while introduction of electron-withdrawing groups, such as acetyl group, halogen (F or Cl) or fused aromatic ring into the basic phenyl moiety, resulted in decreased activity toward the *hpa1* promoter. Considering their strong inhibition, we chose the above three compounds I-9, I-12, and I-13 with the best inhibitory activity for further study.

### Measurement of Growth Curve

**FIGURE 3:**
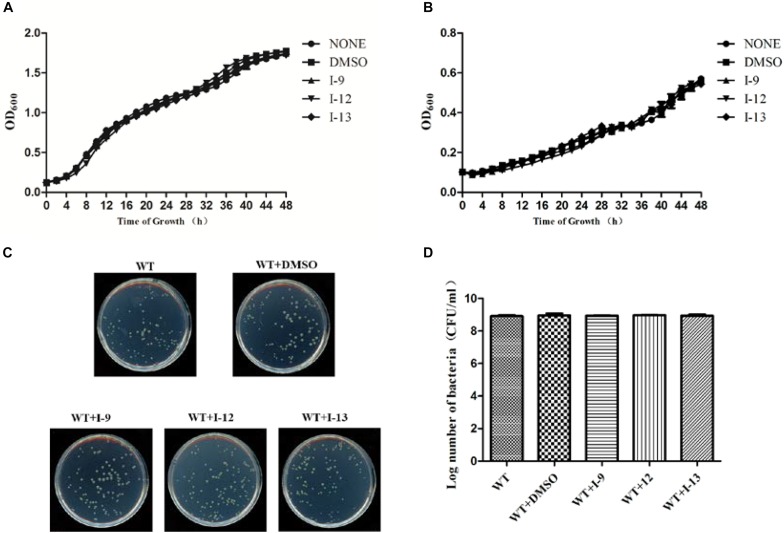
Effects of three compounds (I-9, I-12, and I-13) on bacterial growth. **(A)** The growth rate of *Xanthomonas oryzae* pv. *oryzae* (*Xoo*) PXO99^*A*^ in rich medium (M210) supplemented with DMSO (dimethylsulfoxide) or respectively 100 μM of I-9, I-12, and I-13. **(B)** The growth rate of *Xoo* PXO99^*A*^ in hrp-inducing medium (XOM2 plus 0.5% sucrose) supplemented with DMSO or 100 μM of I-9, I-12, and I-13 respectively. The optical density at 600 nm (OD_600_) of the culture suspensions was recorded every 2 h during the 48 h period. **(C)** Effects of three compounds on bacterial growth. **(D)** Quantify the number of colonies on bacterial growth.

Targeting bacterial virulence factors without influencing bacterial growth is our principle of screening. Although the three compounds I-9, I-12, and I-13 did not show a significant inhibiting effect on bacterial growth in a short period of time, they were still likely to inhibit bacterial growth at different stages of *Xoo*. Therefore, we measured the 48 h growth curve of *Xoo* in both hrp-inducing medium (XOM2, 0.5% sucrose was supplemented to support bacterial growth) and rich medium (M210). Three compounds were added to the media at 100 μM. Compared with the DMSO control, the above three small molecules did not exhibit significant inhibition of bacterial growth. The outcomes showed that these compounds did not affect bacterial growth (Figures 3A,B). At the same time, we used a gradient dilution of *Xoo* cell suspension to perform plate colony counts to analyze whether bacterial growth was affected by the three compounds. At the concentration of 100 μM, the above three compounds did not affect the growth of *Xoo* compared to the DMSO solvent control plate (Figures 3C,D). So, we concentrated on compounds I-9, I-12, and I-13 for the next investigation. To be consistent, all other experiments used a final concentration of 100 μM of each compound.

### Three Compounds Suppress the Hypersensitive Response (HR) Caused by *Xoo* in Tobacco

**FIGURE 4:**
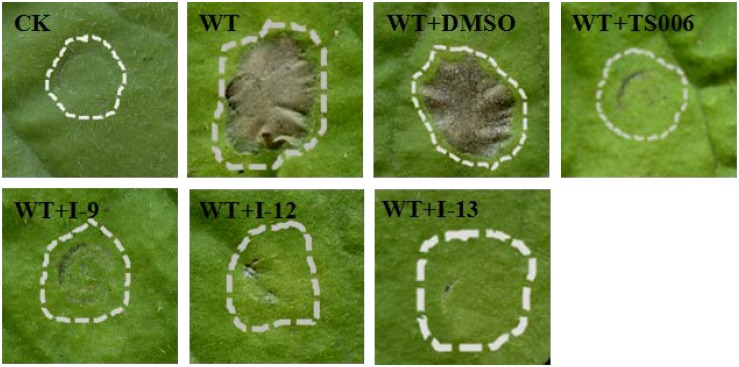
Effects of three compounds (I-9, I-12, and I-13) on the HR induced by *Xoo* on tobacco leaves. Three compounds suppressed HR induced by *Xoo*. TS006 was used as the control. The similar results are representative of at least three independent experiments. HR symptoms appeared at 24 h after inoculation, then the symptoms were recorded by photographing. CK represented DMSO.

*Xoo* cells which had T3SS activity in the bacteria can induce HR on non-host tobacco leaves. It revealed that a functional T3SS in *Xoo* is required for this phenotype. Therefore, we examined the effects of the three compounds I-9, I-12, and I-13 for the HR on non-host tobacco leaves. At a concentration of 100 μM, the above compounds caused significant inhibition of HR (Figure 4). The phenotype of HR showed that the three compounds I-9, I-12, and I-13 could effectively suppress the function of the *Xoo* T3SS at 100 μM. So, we still used the three compounds for the future study, which had significant inhibition of HR and no inhibition of bacterial growth.

### Expression of Representative *hrp*/*hrc* Genes Was Inhibited by Compounds I-9, I-12, and I-13

**FIGURE 5:**
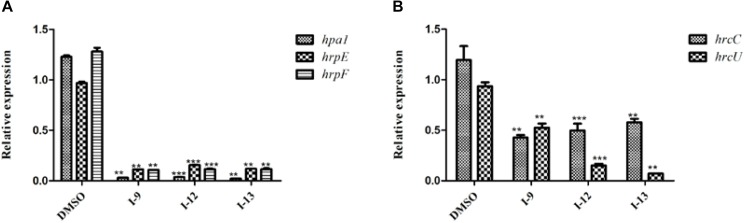
Relative mRNA levels of representative genes in the *hrp* cluster in *Xoo* PXO99^*A*^ incubated with compound I-9, I-12, and I-13 were measured by qRT-PCR. **(A)** Compared with the DMSO control, the mRNA levels of *hap1*, *hrpE* and *hrpF* was reduced significantly after treatment with these inhibitors. **(B)** Compared with the DMSO control, the mRNA levels of two *hrc* genes (*hrcC* and *hrcU*) were also reduced in different extents after treatment with these inhibitors. The DNA gyrase subunit B (*gyrB*) gene was used as the internal control for data analysis. Each experiment had three replicates. ^∗∗^*P* < 0.01, ^∗∗∗^*P* < 0.0001.

**FIGURE 6:**
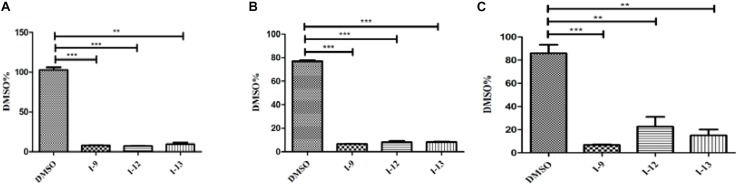
The effects of the three compounds (I-9, I-12, and I-13) on the promoter activity of *hrpG* **(A)**, *hrpX* **(B)** and *hrcT* **(C)** in *Xoo* grown in XOM2 medium supplement with 100 μM of tested compounds. DMSO was used as a solvent control. GFP mean fluorescence intensity (MFI) was determined by flow cytometry. The calculated method was following the formula: DMSO% = 100 × MFI (XOM2 with tested compounds)/MFI (XOM2 with DMSO). Each experiment had three replicates. ^∗∗^*P* < 0.01, ^∗∗∗^*P* < 0.0001.

The above data had shown that the three compounds I-9, I-12 and I-13 inhibited the T3SS gene of *Xoo* without affecting bacterial growth. It was presumed that they may be specific to T3SS. We checked the expression levels of T3SS-related genes in *Xoo* in the presence of three T3SS inhibitors by doing qRT-PCR experiments. In Table 1, the data revealed that compounds I-9, I-12, and I-13 reduced the promoter activity of *hpa1* nearly 98%. The qRT-PCR assays in Figure 5 demonstrated that the mRNA level of *hpa1* was decreased by over 90% in *Xoo* treated with these compounds, which was consistent with the results in Table 1. Then, the expression levels of other key *hrp/hrc* genes were measured, including two *hrp* genes (*hrpE*: encoding the *hrp* pilus protein and *hrpF*: encoding a putative translocon protein) and two hrc genes (*hrcC*: encoding the outer-membrane secretin and *hrcU*: being export apparatus genes) (Tian et al., 2015; Gottig et al., 2018). As we expected, the results in Figure 6 showed that the tested *hrp/hrc* genes in the mRNA levels were reduced by different degrees when comparing with DMSO control.

### The Three Compounds Affect the Expression of Regulatory Genes *hrpG* and *hrpX*

*HrpG* and *hrpX* controlled the expression of the *Xanthomonas* T3SS with the regulatory cascade (Gurlebeck et al., 2006). Although we found that treatment with the compounds I-9, I-12, and I-13 could reduce in mRNA levels in representative *hrp/hrc* genes in *Xoo*, it was still essential to demonstrate whether the expression of *hrpG* and *hrpX* was affected. The mRNA levels of *hrpG* and *hrpX* were tested when *Xoo* cells were treated with each inhibitor, and the mRNA level of *hrpX* and *hrpG* was reduced by over 80% (Figures 6A,B). These results showed that the inhibitory effect of I-9, I-12, and I-13 on T3SS gene expression of *Xoo* was really through the HrpG-HrpX regulatory cascade.

The transcriptional regulator HrpX controlled most genes in the *hrp* cluster, which discriminated the PIP-box (TTCGC-N15-TTCGC) in the promoter region, such as that of *hpa1* in Supplementary Figure S2. To examine the impact of the compounds on the transcription of T3SS genes in *Xoo*, the promoter for the *hrcT* operon (named as P_*hrcT*_) carrying a perfect PIP-box in Supplementary Figure S3 was used to demonstrate the impact of the compounds I-9, I-12, and I-13 on the activity of P_*hrcT*_ by using FACS assays. The results in Figure 6C showed that P_*hrcT*_ activity reduced after treatment with I-9, I-12, and I-13 by comparing with the DMSO control (DMSO%). These results were similar to that of on the *hpa1* promoter, which showed that promoters carrying a PIP-box may be the key targets of these inhibitors. The results also demonstrated that HrpX played a core role in the regulation of the inhibitory function.

### Three Compounds Do Not Suppress the Expression of Other Virulence Factors of *Xoo* and Swimming Motility

**FIGURE 7:**
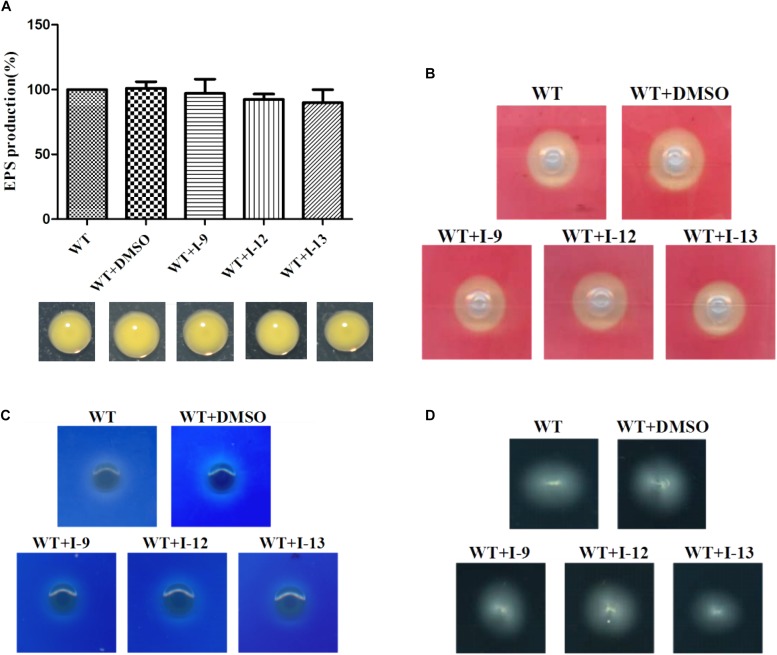
**(A)** Secretion of exopolysaccharide (EPS) in *Xoo* wild-type and that of treatment with I-9, I-12, or I-13 compounds had no significant difference. From left to right, it provided WT, WT + DMSO, WT + I-9, WT + I-12, and WT + I-13. Error bars represent standard deviation from three repeats. **(B)** Detection of cellulase secreted by *Xoo* grown in PSA containing 0.5% carboxymethyl cellulose for 24 h. Pale-yellow clear zones showed no difference between *Xoo* wild-type and that of treatment with I-9, I-12, or I-13 compounds. **(C)** Detection of xylanase secreted by *Xoo* grown in PSA 0.2% RBB-xylan for 48 h. Both *Xoo* wild-type and that of treatment with I-9, I-12, or I-13 compounds were appeared as the white clear zones among a blue background. **(D)** Detection of swimming motility on 0.3% soft agar plates supplemented with I-9, I-12, or I-13 compounds at 28°C after 72 h.

*Xoo* had different virulence factors including T3SS, extracellular enzymes and EPS. The objective of our work was to find compounds that targeted T3SS without affecting the function of other virulence factors. Therefore, we checked representative virulence factor such as EPS, extracellular cellulase, extracellular xylanase. Compared with that of the wild-type, the *Xoo* cells which were treated with I-9, I-12, and I-13 conveyed no significant difference in the surface of the colony (Figure 7A).

After observing the phenotype, the EPS production was quantified from wild-type and the *Xoo* cells which were treated with I-9, I-12, and I-13 (Figure 7A). The results clearly showed that EPS production had no significant difference. At the same time, we did not find changes in the secretion of extracellular cellulase or xylanase between the wild-type and the *Xoo* cells which were treated with I-9, I-12, and I-13 (Figures 7B,C).

Motility is considered as an important attribute that enables pathogens to reach specific sites in the host (Buttner and Bonas, 2010). We also checked whether our compounds to see if they influenced the swimming motility of *Xoo*. The results did not show any substantial reduction in diameter (Figure 7D).

### Three Compounds Suppress the Symptoms of *Xoo* on Rice

**FIGURE 8:**
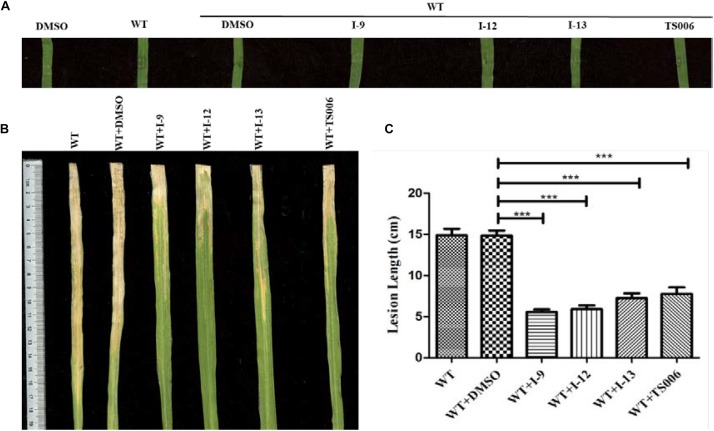
**(A)** The effect of I-9, I-12, and I-13 on the water-soaking symptoms caused by *Xoo* wild-type on IR24 seedling. From left to right, it provided pure DMSO, WT, WT + DMSO, WT + I-9, WT + I-12, and WT + I-13.The disease symptoms **(B)** and lesion lengths **(C)** of *Xoo* wild-type on adult plants of rice cultivar IR24 were reduced after supplement with 100 μM of I-9, I-12, and I-13. At least three experiment tests had similar results. Asterisks indicate statistically significant differences. ^∗∗∗^*P* < 0.0001.

The ultimate aim of this work was to examine that the selected inhibitors could suppress disease symptoms on host. We used the seedlings of susceptible rice (cultivar IR24) to assess the symptoms of water-soaked lesions, which were induced by *Xoo* PXO99^*A*^ after penetrating bacterial cells into the leaves. The water soaking symptoms on seedlings (Figure 8A) were reduced by adding compounds I-9, I-12, I-13, and TS006 to the bacterial cells. The yellowish disease symptoms on adult IR24 plants (Figure 8B) were also weakened significantly. Furthermore, in contrast to DMSO (14.86 cm), treatments of I-9 (5.56 cm), I-12 (5.90 cm), I-13 (7.25 cm) and TS006 (7.78 cm) could significantly reduce the lesion lengths (Figure 8C). TS006 was used as the control.

*In vivo* protection activity was evaluated and the results indicated that the test compounds exerted better or similar protective activity (I-9: 58.93%, I-12: 58.02%, I-13: 50.74%, Table 2) against rice bacterial leaf blight compared with TS006 (49.37%), commercial drugs bismerthiazol (50.40%) and thiodiazole copper (46.87%) in greenhouse test. Meanwhile, the test compounds were found safe to the plants.

**TABLE 2:** Protection activity of compounds against rice bacterial leaf blight under greenhouse conditions at 200 μg/mL.

**Treatment**	**14 days after inoculation**		
	**Morbidity (%)**	**Disease index (%)**	**Control efficiency (%)**
I-9	100	36.10	58.93^a^
I-12	100	36.90	58.02^a^
I-13	100	43.30	50.74^c^
TS006	100	44.50	49.37^b^
Bismerthiazol	100	43.60	50.40^c^
Thiodiazole copper	100	46.70	46.87^d^
CK	100	87.90	–

*The letters a–d denoted the results of difference significance analysis. The different letter indicates the values of activity with significant difference among different treatment groups (*P* < 0.05, Fisher’s LSD multiple comparison test).*

### Effects of Three T3SS Inhibitors on *Xcc*

**FIGURE 9:**
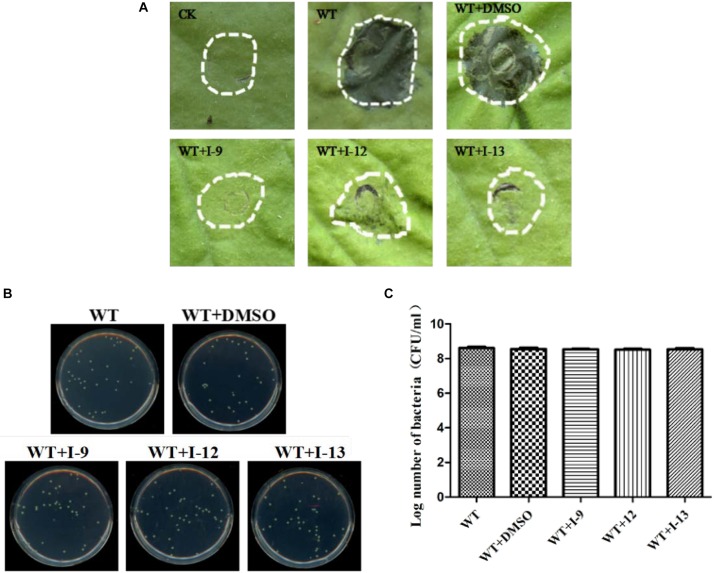
**(A)** Effects of three compounds (I-9, I-12, and I-13) on the HR induced by *Xcc* on tobacco leaves. Three compounds suppressed HR induced by *Xcc*. The similar results are representative of at least three independent experiments. **(B)** Effects of three compounds on bacterial growth. **(C)** Quantify the number of colonies on bacterial growth.

**FIGURE 10:**
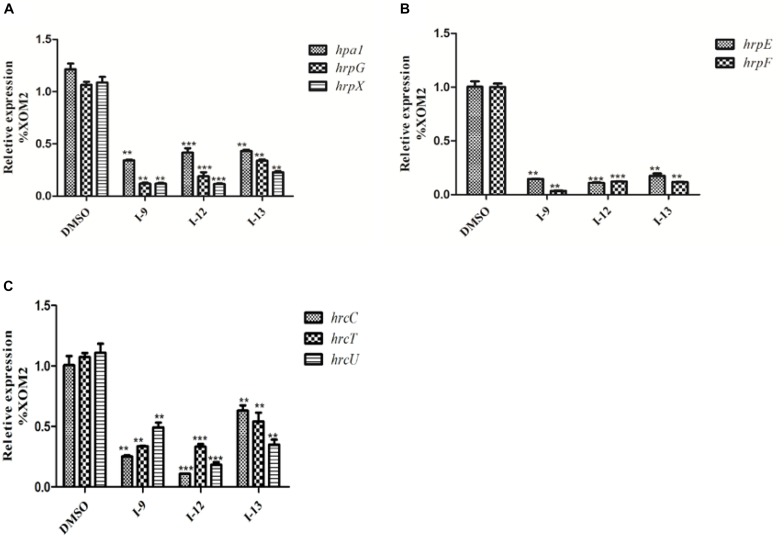
Relative mRNA levels of representative genes in the *hrp* cluster in *Xcc*8004 incubated with compound I-9, I-12, and I-13 were measured by qRT-PCR. **(A)** Compared with the DMSO control, the mRNA levels of *hap1, hrpG*, and *hrpX* were dropped remarkably after adding these inhibitors. **(B)** Compared with the DMSO control, the mRNA levels of *hrpE* and hrpF genes were dropped considerably after adding these inhibitors. **(C)** Compared with the DMSO control, the mRNA levels of three *hrc* genes (*hrcC*, *hrcT*, and *hrcU*) were also dropped in different degree after adding these inhibitors. The DNA gyrase subunit B (*gyrB*) gene was used as the internal control for data analysis. Each experiment had three replicates. ^∗∗^*P* < 0.01, ^∗∗∗^*P* < 0.0001.

Considering that T3SS is a very conserved key virulence factor in gram-negative pathogenic bacteria (Beattie and Lindow, 1995), this intrigued us to further find whether our inhibitors could inhibit T3SS of other bacteria. *Xanthomonas campestris* pv. *campestris* (*Xcc*) which may have a similar phenotype with *Xoo*, belongs to the *Xanthomonas* genus, which cause huge harm to agricultural production (Vauterin et al., 1995). *Xcc* is a model bacterium for studying molecular plant pathology and one of the most serious diseases causing cruciferous plant diseases (Qian et al., 2005). Due to the conservation of T3SS, we wanted to investigate whether the three compounds would have the same effect on *Xcc* T3SS. According to reports (Qian et al., 2005), the *hrp* gene in *Xcc* is positively regulated by *hrpG* and *hrpX* in varying degrees. Hpa1 in *Xcc* could also trigger HR on tobacco. We used this feature to construct a screening system. To screen compounds that inhibited the expression of the *Xcc* T3SS, we used a FACS-Caliber flow cytometer as previously described to analyze the promoter activity of *Xcc hpa1* in Supplementary Figure S9. The results manifested the three compounds inhibited the *Xcc hpa1* promoter activities by at least 60% by comparison with the DMSO (Table 3). Then, we performed an experiment to examine the effects of three compounds for the HR on tobacco leaves. The results revealed that the three compounds I-9, I-12, and I-13 could effectively suppress phenotype of HR on tobacco leaves (Figure 9A). The feature suggested the above three compounds might suppress T3SS of *Xcc*. Meanwhile, we checked *Xcc* growth by using a gradient dilution of *Xcc* cell suspension to perform plate colony counts. The results showed that the above three compounds did not affect the growth of *Xcc* when compared to the DMSO solvent control (Figures 9B,C). The above data had shown that the three compounds inhibited the T3SS function and did not affect the growth of *Xcc*. Then we ensure the expression levels of some gene related to T3SS in *Xcc* by performing qRT-PCR experiments. The qRT-PCR assays in Figure 10 showed that the mRNA level of *hpa1* was dropped remarkably in *Xcc* by adding these compounds. The expression levels of other key *hrp/hrc* genes were reduced in different degrees by comparing with DMSO control.

**TABLE 3:** Screening for inhibitors of *Xcc* T3SS by fluorescence-activated cell sorting assays.

**Compound**	**Avg MFI ± SD^a^**	**DMSO%^b^**	**Inhibition rate% (100% – DMSO%)**
			
DMSO	2726.15 ± 80.35		
I-9	783.27 ± 83.82	29.83	70.17
I-12	938.43 ± 10.10	35.73	64.27
I-13	1011.50 ± 204.70	38.52	61.48

*^*a*^Green fluorescent protein (GFP) and mean fluorescence intensity (MFI) were measured for gated populations of bacterial cells by flow cytometry (bacterial cells cultured in XCM2 with DMSO or XCM2 added with 100 μM of each compound). Values are representative of at least three independent experiments, and three replicates were done for each experiment. ^*b*^The calculated method was following the formula: DMSO% = 100 × MFI (XCM2 with tested compounds)/MFI (XCM2 with DMSO).*

**FIGURE 11:**
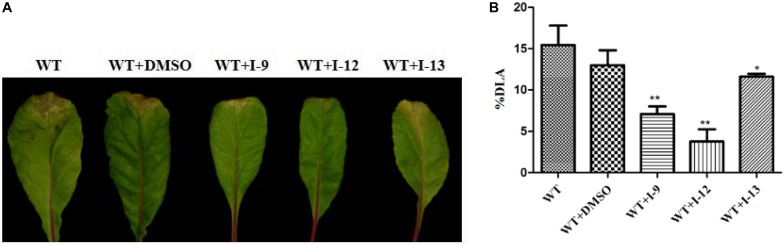
The effect of I-9, I-12, and I-13 on the “V” shaped yellow disease symptoms caused by *Xcc* 8004 wild-type on Chinese radish. **(A)** From left to right, it provided WT, WT + DMSO, WT + I-9, WT + I-12, and WT + I-13. **(B)** The percentage of diseased leaf area (%DLA) was quantification of the reduction in lesion area by the selected compounds. %DLA = area covered by lesions/whole leaf area × 100.

We finally accomplished the pathogenicity assays on the host plants. The “V” shaped yellow disease symptoms on plants were reduced to different extents by adding compounds I-9, I-12, and I-13 to the bacterial cells (Figure 11). The above results indicated that the three compounds may inhibit *Xcc* T3SS. It increased the broad spectrum of the three compounds.

## Conclusion

Currently, the emergence of the resistance of *Xoo* against traditional bactericides should serve as a wakeup call for developing new anti- *Xoo* (and other pathogen) drugs. To find new solutions, most studies have focused on T3SS inhibitors, which is a novel type of antibiotic. *Xoo* and many other gram-negative bacteria invade host plant cells by using type three secretion systems. T3SS is highly conserved among most gram-negative pathogenic bacteria but not necessary for bacterial growth and survival. In order to develop new T3SS inhibitors, we have screened a series of ethyl 2-nitro-3-arylacrylates, synthesized by a feasible method. The bioassay results showed that the three of these compounds I-9, I-12, and I-13 inhibited the promoter activity of *hpa1* in *Xoo* significantly, and they can reduce HR without affecting *Xoo* bacterial growth or survival. HrpG and HrpX are two key regulatory proteins in *Xanthomonas*, and their *hrp* genes expressions are mainly regulated by HrpX-HrpG pathway. Our results showed that three potential T3SS inhibitors reduced the expression of *hrp*/*hrc* gene probably through HrpX-HrpG pathway. Meanwhile, three compounds could reduce the disease symptoms of *Xoo* on the rice. We also expanded the therapeutic spectrum of our compounds. Our results found that three compounds could reduce some special genes expression in *Xcc* without affecting bacterial growth. I-9 and I-12 show a better ability to attenuate lesions on the Chinese radish plants than I-13. Three compounds might be applied to inhibit infection in agricultural production. However, before applying these inhibitors, a major problem is that exact mechanisms remain unclear, which will need to be explored in our next work.

## Data Availability

The raw data supporting the conclusions of this manuscript will be made available by the authors, without undue reservation, to any qualified researcher.

## Author Contributions

Z-NC and GS conceived and designed the experiments. SJ and HL performed the experiments. SJ, HL, and WA analyzed the data. SJ, HL, WA, XX, and Z-NC collaborated in the discussion and interpretation of results. SJ, WA, and Z-NC wrote the manuscript.

## Conflict of Interest Statement

The authors declare that the research was conducted in the absence of any commercial or financial relationships that could be construed as a potential conflict of interest.
