# Supplementary material for: Discovery of Ethyl 2-Nitro-3-Arylacrylates Molecules as T3SS Inhibitor Reducing the Virulence of Plant Pathogenic Bacteria Xanthomonas
Source: Front Microbiol. 2019 Aug 20;10:1874. doi: 10.3389/fmicb.2019.01874 (PMC6710329; doi:10.3389/fmicb.2019.01874)
Supplement: Supplementary file 1 [file Table_1.DOCX]

**Discovery of ethyl 2-nitro-3-arylacrylates molecules as T3SS inhibitor reducing the virulence of plant pathogenic bacteria *Xanthomonas***

*Shan Jiang^1,2^***^†^***, Hui Li^2^***^†^***, Wasim Ahmed^1^***^†^***, Xuwen Xiang^1^,*

*Gaopeng Song^2,^*, Zi-Ning Cui^1,^**

^1^State Key Laboratory for Conservation and Utilization of Subtropical Agro-bioresources, Integrative Microbiology Research Centre, Guangdong Province Key Laboratory of Microbial Signals and Disease Control, South China Agricultural University, Guangzhou 510642, China, ^2^College of Materials and Energy, South China Agricultural University, Guangzhou, 510642, China

*Correspondence: [ziningcui@scau.edu.cn (Z. C.);](mailto:ziningcui@scau.edu.cn;) vinsin1021@126.com (G. S.); Tel.: +86-20-85288229; Fax: +86-20-85288229

**^†^** These authors contributed equally to this paper.

**Table S1.** Strains plasmids and primers used in this study

| **Strains, plasmids and primers** | **Relevant characteristics** | **Reference or source** |
| --- | --- | --- |
| **Strains** |  |  |
| *Xanthomonas oryzae* pv. *Oryzae*PXO99^A^ | Wild-type strain, Philippine race 6, Cp^r^ | Dr.Chenyang He |
| *Xanthomonas campestris pv. Campestris 8004* | Wild-type strain，Rif ^r^ | Lab collection |
| **Plasmids** |  |  |
| pPROBE-AT | Promoter-probe vector, Ap^r^ | Lab collection |
| pPhpa1 pProbe-AT in PXO99^A^ | Derivative with PCR fragment containing *hpa1* promoter region, Ap^r^ | Dr.Chenyang He |
| pPhpa1 pProbe-AT in 8004 | Derivative with PCR fragment containing *hpa1* promoter region, Ap^r^ | Dr.Chenyang He |
| pPhrpG pProbe-AT in PXO99^A^ | Derivative with PCR fragment containing *hrpG* promoter region, Ap^r^ | Dr.Chenyang He |
| pPhrpX pProbe-AT in PXO99^A^ | Derivative with PCR fragment containing *hrpX* promoter region, Ap^r^ | Dr.Chenyang He |
| pPhrcT pProbe-AT in PXO99^A^ | Derivative with PCR fragment containing *hrcT* promoter region, Ap^r^ | Dr.Chenyang He |
| **Primers** | **Sequences 5’ - 3’** | **Source** |
| gyrB-F | GCGACGATTTCCACTACG | This study |
| gyrB-R | CCTTCTGCGGGATCTTATTG | This study |
| *Xcc* hrpG-F | CAAGGATCGGCATTCGTACTGAC | This study |
| *Xcc* hrpG-R | CCAGGCGAGCAGGGAACTG | This study |
| *Xcc* hrpX-F | TCTGCCTCTTCCTGATACTCCAG | This study |
| *Xcc* hrpX-R | GCGATCTCTGCGTTGTCTTACG | This study |
| *Xcc* hrcC-F | AGATTCTGAGCACCGAGCCATC | This study |
| *Xcc* hrcC-R | CCTGTCCGCCAGCCAATCC | This study |
| *Xcc* hrcT-F | CTGCTGCTGGAAGTGGAGGAAG | This study |
| *Xcc* hrcT-R | GGTGCTGGTGCTGGTGGATC | This study |
| *Xcc* hrpE-F | ACAATCACACTGCCACTCAGACC | This study |
| *Xcc* hrpE-R | GCCTGCCTTGCCGATGAATTTG | This study |
| *Xcc* hrpF-F | TGAGACGCCATCGCAAAGAAC | This study |
| *Xcc* hrpF-R | GGCACGCTGACGCAATCC | This study |
| *Xcc* hrcU-F | ACTGGAACTGCGTGCCTATGC | This study |
| *Xcc* hrcU-R | ACCGCTGCCTTCATCGTTGTC | This study |

Ap^r^, ampicillin resistance; Cp^r^, cephalexin resistance;


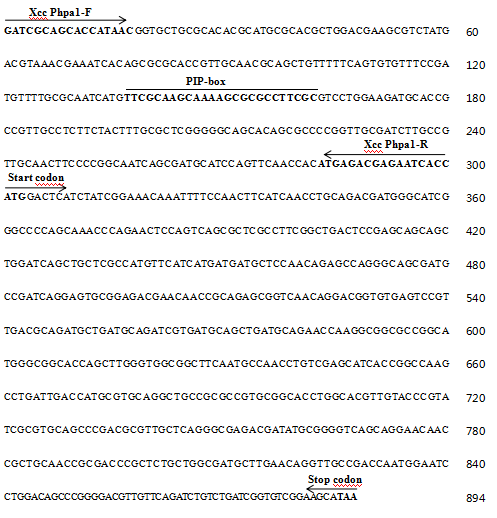


**Figure S1.** DNA sequence of xcc 8004 *hpa1* gene and its promoter region. Sequences for a PIP box, start codon, stop codon and the primers used for amplification and rif of the promoter region are indicated as bold fonts


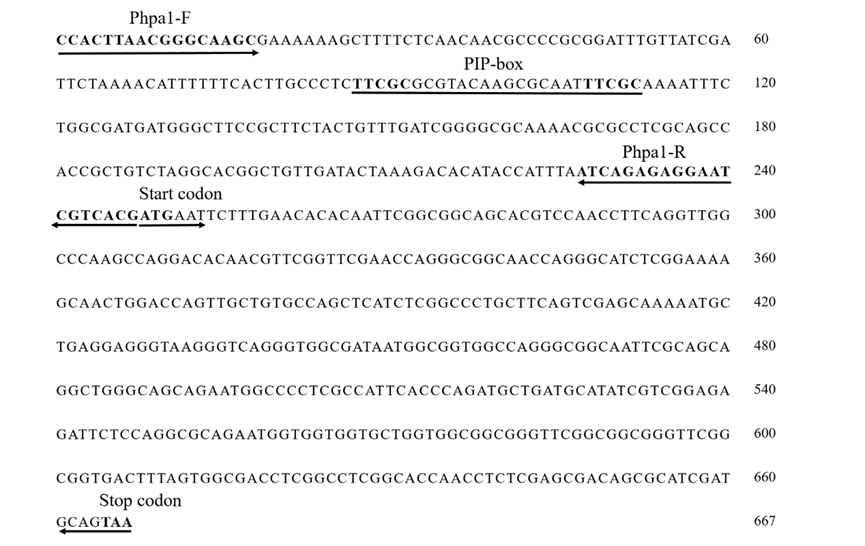


**Figure S2.** DNA sequence of *Xoo* *hpa1* gene and its promoter region. Sequences for a PIP box, start codon, stop codon and the primers used for amplification of the promoter region are indicated as bold fonts.


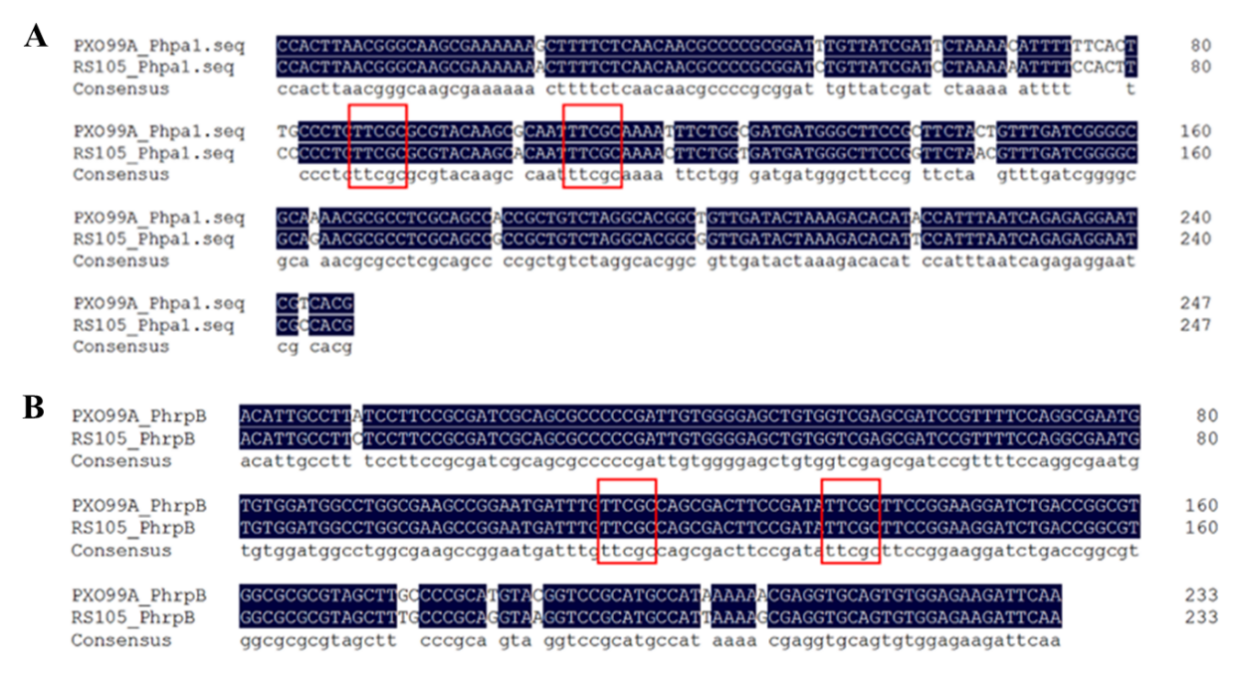


**Figure S3.** Sequence alignment of promoter regions of *hpa1* (A), *hrcT* (B) between *Xoo*

***Synthesis of title compounds* (I-11 to I-24).**

**Scheme 1.** The synthetic route of the title compounds I-11 to I-24. Reagents and conditions: (a) halohydrocarbon, K_2_CO_3_, DMF or Ac_2_O, DMAP, pyridine; (b) NO_2_CH_2_COOEt, (CH_3_)_2_NH∙HCl, KF, toluene, reflux.

**Figure S4.** The chemical structures of title compounds I-1 to I-24.

To the substituted benzaldehydes (0.15 mol) dissolved in anhydrous toluene was added ethyl nitroacetate (1.2 mol), dimethylamine hydrochloride (1.8 mol), and potassium fluoride (0.15 mmol). Reaction mixture was then refluxed at 120 °C under argon. After 24 hours, the reaction mixture was cooled to room temperature and concentrated under reduced pressure to obtain the residue which was diluted with dichloromethane. The organic phase was washed with water, brine, and dried over anhydrous Na_2_SO_4_. It was filtered and concentrated to dryness *in vacuo*. The residue was purified by flash chromatography (20:1, petroleum ether: EtOAc) to give I-11 to I-24 as a light yellow solid, respectively.

**(*Z* and *E*) Ethyl-3-(3-(cyclopentyloxy)-4-methoxyphenyl)-2-nitroacrylate (I-11)**

Yellow solid, yield 70%; ^1^ H NMR (CDCl_3_): *δ* *8.02 (s, 1H, C=C*H*), 7.43 (s, 1H, C=C*H*), *7.15 (dd, 1H, *J* = 8.4, 2.1 Hz, Ar-H-6), *7.06 (d, 1H, *J* = 2.1 Hz, Ar-H-2), 7.04 (dd, 1H, *J* = 8.4, 2.1 Hz, Ar-H-6), 6.91 (d, 1H, *J* = 2.2 Hz, Ar-H-2), *6.90 (d, 1H, *J* = 8.5 Hz, Ar-H-5), 6.86 (d, 1H, *J* = 8.4 Hz, Ar-H-5), 4.67-4.75 (m, 2H, OC*H*, *OC*H*), *4.45 (q, 2H, *J* = 7.1 Hz, OC*H*_2_CH_3_), 4.37 (q, 2H, *J* = 7.1 Hz, OC*H*_2_CH_3_), *3.91 (s, 3H, OC*H*_3_), 3.89 (s, 3H, OC*H*_3_), 1.77-1.92 (m, 8H, 4×C*H*_2_), *1.58-1.68 (m, 8H, 4×C*H*_2_), *1.39 (t, 3H, *J* = 7.2 Hz, OCH_2_C*H*_3_), 1.36 (t, 3H, *J* = 7.1 Hz, OCH_2_C*H*_3_); ^13^C NMR (CDCl_3_): *δ* 159.6, 153.6, 148.1, 138.1, 133.0, 125.3, 121.3, 113.9, 111.5, 80.6, 62.8, 56.0, 32.7 (two), 24.2 (two), 14.1; HRESIMS calcd for C_17_H_21_NO_6_Na 358.1267; found 358.11269.

**(*Z* and *E*) Ethyl-3-(3-(benzyloxy)-4-methoxyphenyl)-2-nitroacrylate (I-12)**

Yellow solid, yield 65%; ^1^H NMR (600 MHz, CDCl_3_): *δ* 7.97 (s, 1H, C=C*H*), *7.45 (s, 1H, C=C*H*) ,7.44 (t, 3H, *J* = 7.1 Hz, Ar-H), 7.38 (td, 1H, *J* = 7.3, 1.8 Hz, Ar-H), *7.30-7.35 (m, 2H, Ar-H), 7.18 (dd, 1H, *J* = 8.4, 2.1 Hz, Ar-H-6), 7.10 (d, 1H, *J* = 2.1 Hz, Ar-H-2), *7.07 (dd, 1H, *J* = 8.5, 2.1 Hz, Ar-H-6), 6.92-6.94 (m, 2H, Ar-H), 6.89 (d, 1H, *J* = 8.5 H, Ar-H-5), 5.13 (s, 2H, Bn-C*H*_2_), *5.12 (s, 2H, Bn-C*H*_2_), 4.35 (q, 4H, *J* = 7.1Hz, OC*H*_2_CH_3_), 3.94 (s, 3H, OC*H*_3_), *3.92 (s, 3H, OC*H*_3_), 1.34 (t, 6H, *J* = 7.1 Hz, C*H*_3_); ^13^C NMR (150 MHz, CDCl_3_): *δ* 161.7, 159.6 , 153.8, 153.4, 148.6, 148.5, 140.0, 138.2, 136.8, 136.2, 132.8 (two), 128.7 (two), 128.6 (two), 128.2 , 128.1, 127.5 (two), 127.3 (two), 126.7, 125.8, 121.5, 115.0, 114.0, 111.8 , 111.7 , 71.1, 71.0, 63.0, 62.8, 56.1, 56.0, 14.1, 13.8; HRESIMS calcd for C_19_H_19_NO_6_Na 380.3518; found 380.3520.

**(*Z* and *E*) Ethyl-3-(4-cyclopentyloxy phenyl)-2-nitroacrylate (I-13)**

Yellow solid, yield 61%; ^1^H NMR (600 MHz, CDCl_3_): *δ* 8.03 (s, 1H, C=C*H*), 7.46 (d, *J* = 8.9 Hz, 2H, Ar-H-3, Ar-H-5), *7.45 (s, 1H, C=C*H*), *7.36 (d, *J* = 8.9 Hz, 2H, Ar-H-3, Ar-H-5), 6.90 (d, *J* = 8.9 Hz, 2H, Ar-H-2, Ar-H-6), *6.87 (d, *J* = 8.9 Hz, 2H, Ar-H-2, Ar-H-6), 4.78-4.87 (m, 2H, H-1’, *H-1’), 4.46 (q, *J* = 7.1 Hz, 2H, OC*H*_2_CH_3_), *4.36 (q, *J* = 7.1 Hz, 2H, OC*H*_2_CH_3_), 1.89-1.99 (m, 4H, 4×C*H*_2_), 1.75-1.89 (m, 6H, 3×C*H*_2_), 1.58-1.69 (m, 2H, C*H*_2_), 1.38 (t, *J* = 7.2 Hz, 3H, C*H*_3_), *1.35 (t, *J* = 7.1 Hz, 3H, C*H*_3_); ^13^C NMR (150 MHz, CDCl_3_): *δ* 162.1, 161.9, 161.7, 159.7, 137.9, 136.7, 133.0 (two), 132.7 (two), 132.2 (two), 120.8, 120.7, 116.3 (four), 115.6, 78.0, 79.9, 62.9, 62.7, 32.8 (two), 24.0 (four), 14.1, 13.8; HRESIMS calcd for C_16_H_19_NO_5_Na 328.1161; found 328.1165.

**(*Z* and *E*) Ethyl-3-{(3, 4-di-cyclopentyloxy)-phenyl}-2-nitroacrylate (I-14)**

Yellow solid, yield 55%; ^1^H NMR (600 MHz, CDCl_3_): *δ* 8.00 (s, 1H, C=C*H*), *7.41 (s, 1H, C=C*H*), 7.12 (dd, *J* = 8.4, 2.2 Hz, 1H, Ar-H-6), 7.06 (d, *J* = 2.2 Hz, 1H, Ar-H-2), *7.01 (dd, *J* = 8.4, 2.2 Hz, 1H, Ar-H-6), *6.92 (d, *J* = 2.2 Hz, 1H, Ar-H-2), 6.88 (d, *J* = 8.4 Hz, 1H, Ar-H-5), *6.85 (d, *J* = 8.5 Hz, 1H, Ar-H-5), 4.78-4.88 (m, 2H, 2×OC*H*), *4.64-4.72 (m, 2H, 2×OC*H*), 4.44 (q, *J* = 7.1 Hz, 2H, OC*H*_2_CH_3_), *4.36 (q, *J* = 7.1 Hz, 2H, OC*H*_2_CH_3_), 1.74-1.94 (m, 16H, 8×C*H*_2_), 1.62 (m, 8H, 4×C*H*_2_), 1.38 (t, *J* = 7.1 Hz, 3H, CH_2_C*H*_3_), *1.35 (t, *J* = 7.1 Hz, 3H, CH_2_C*H*_3_); ^13^C NMR (150 MHz, CDCl_3_): *δ* 161.9, 159.7, 153.6, 153.0, 148.9, 148.8, 138.0, 137.0, 133.0 (two), 126.6, 125.4, 121.2, 121.1, 117.6, 116.5, 115.0, 114.9, 81.5, 81.3, 80.9, 80.8, 62.9, 62.7, 32.9 (two), 32.8 (two), 32.7 (two), 32.6 (two), 24.0 (eight), 14.1, 13.8; HRESIMS calcd for C_21_H_27_NO_6_Na 412.4378; found 412.4381.

**(*Z* and *E*) Ethyl-3-{(3, 4-di-pentyloxy)-phenyl}-2-nitroacrylate (I-15)**

Yellow solid, yield 60%; ^1^H NMR (600 MHz, CDCl_3_): *δ* 8.01 (s, 1H, C=C*H*), *7.43 (s, 1H, C=C*H*), 7.14 (d, 1H, *J* = 8.4 Hz, Ar-H-5), *7.03-7.05 (m, 2H, Ar-H-5, Ar-H-2), 6.91 (s, 1H, Ar-H-2), 6.89 (d, 1H, *J* = 8.5 Hz, Ar-H-6), *6.86 (d, 1H, *J* = 8.4 Hz, Ar-H-6), 4.44 (q, 2H, *J* = 7.1 Hz, OC*H*_2_CH_3_), 4.32-4.38 (m, 4H, 2×OC*H*_2_CH_2_), *4.02-4.07 (m, 4H, 2×OC*H*_2_CH_2_), *3.96 (q, 2H, *J* = 6.1 Hz, OC*H*_2_CH_3_), 1.80-1.86 (m, 8H, 2×OCH_2_C*H*_2_, 2×OCH_2_C*H*_2_*), 1.42-1.49 (m, 6H, 3×C*H*_2_), 1.39 (dt, *J* = 14.8, 7.4 Hz, 10H, 5×C*H*_2_), 1.33 (t, 6H, *J* = 7.0 Hz, C*H*_3_, *C*H*_3_), 0.94 (t, 12H, *J* = 6.0 Hz, 2×C*H*_3_, 2×C*H*_3_*); ^13^C NMR (150 MHz, CDCl_3_): *δ* 161.9, 161.7, 153.5, 153.0, 150.0, 149.2, 138.0, 137.0, 133.0 (two), 126.5, 125.3, 121.3, 121.2, 114.5, 113.8, 112.8, 112.7, 69.3, 69.2, 69.1, 69.0, 62.9, 62.7, 28.8 (two), 28.7 (two), 28.2 (three), 28.1, 22.5, 22.4 (two), 22.4, 14.1, 14.0 (two), 13.9 (two), 13.8; HRESIMS calcd for C_21_H_31_NO_6_Na 416.4698; found 416.4702.

**(*Z* and *E*) Ethyl-3-(3-methoxy-4-(pentyloxy)phenyl)-2-nitroacrylate( I-16)**

Yellow solid, yield 67%; ^1^H NMR (600 MHz, CDCl_3_): *δ* 8.03 (s, 1H, C=C*H*), *7.44 (s, 1H, C=C*H*), 7.16 (d, 1H, *J* = 8.4 Hz, Ar-H-5), *7.06 (d, 1H, *J* = 8.4 Hz, Ar-H-5), 7.05 (s, 1H, Ar-H-3), *6.91 (s, 1H, Ar-H-3), 6.90 (d, 1H, *J* = 6.8 Hz, Ar-H-6), *6.87 (d, 1H, *J* = 8.4 Hz, Ar-H-6), 4.45 (q, 2H, *J* = 7.1 Hz, OC*H*_2_CH_3_), *4.37 (t, 2H, *J* = 7.1 Hz, OC*H*_2_CH_3_), 4.08-4.12 (m, 2H, OC*H*_2_CH_2_), 4.06 (t, *J* = 6.7 Hz, 2H, OC*H*_2_CH_2_), 3.86 (s, 3H, OC*H*_3_), *3.84 (s, 3H, OC*H*_3_), 1.83-1.89 (m, 4H, 2×C*H*_2_), 1.38-1.48 (m, 8H, 4×C*H*_2_), 1.36 (t, 6H, *J* = 7.2 Hz, 2×C*H*_3_), 0.93 (t, 6H, *J* = 6.9 Hz, 2×C*H*_3_); ^13^C NMR (150 MHz, CDCl_3_): *δ* 161.9, 159.6, 152.9, 152.8, 149.7 (two), 138.2, 137.1, 133.0 (two), 126.6, 125.3, 121.3 (two), 112.3 (two), 111.9 (two), 69.2, 69.1, 63.0, 62.8, 56.0 (two), 28.6 (two), 28.0 (two), 22.4(two), 14.1, 14.0 (two), 13.9; HRESIMS calcd for C_17_H_23_NO_6_Na 360.1423; found 360.1425.

**(*Z* and *E*) Ethyl-3-(3-pentyloxy-4-methoxyphenyl)-2-nitroacrylate (I-17)**

Yellow solid, yield 62%; ^1^H NMR (600 MHz, CDCl_3_): *δ* 8.02 (s, 1H, C=C*H*), *7.44 (s, 1H, C=C*H*), 7.16 (d, 1H, *J* = 8.3 Hz, Ar-H-5), *7.07 (d, 1H, *J* = 8.5 Hz, Ar-H-5), 7.02 (s, 1H, Ar-H-2), *6.92 (s, 1H, Ar-H-2), 6.88 (d, 1H, *J* = 8.4 Hz, Ar-H-6), 4.45 (q, 2H, *J* = 7.1 Hz, OC*H*_2_CH_3_), *4.35-4.40 (m, 2H, OC*H*_2_CH_3_), 4.04 (t, 2H, *J* = 6.7 Hz, OC*H*_2_CH_2_), *3.98 (t, 2H, *J* = 6.7 Hz, OC*H*_2_CH_2_), 3.93 (s, 3H, OC*H*_3_), *3.91 (s, 3H, OC*H*_3_), 1.80-1.91 (m, 4H, OCH_2_C*H*_2_, *OCH_2_C*H*_2_), 1.39-1.50 (m, 8H, 4×C*H*_2_), 1.36 (t, 6H, *J* = 7.3 Hz, OCH_2_C*H*_3_, OCH_2_C*H*_3_*), 0.94 (t, 6H, *J* = 6.8 Hz, C*H*_3_, C*H*_3_*); ^13^C NMR (150 MHz, CDCl_3_): *δ* 161.9, 161.8, 153.5, 153.1, 149.2, 148.9, 138.2, 136.9, 135.1, 132.9, 125.2, 124.5, 121.5, 121.4, 113.7, 112.9, 111.9, 111.5, 69.1, 69.1, 62.9, 62.8, 56.1, 56.0, 28.7, 28.6, 28.0 (two), 22.4 (two), 14.1, 14.0, 13.9, 13.8; HRESIMS calcd for C_17_H_23_NO_6_Na 360.1423; found 360.1425.

**(*Z* and *E*) Ethyl-3-(4-pentyloxyphenyl)-2-nitroacrylate (I-18)**

Yellow solid, yield 65%; ^1^H NMR (600 MHz, CDCl_3_): *δ* 8.03 (s, 1H, C=C*H*), 7.47 (d, 2H, *J* = 8.7 Hz, Ar-H-3, Ar-H-5), *7.45 (s, 1H, C=C*H*), *7.37 (d, 2H, *J* = 8.5 Hz, Ar-H-3, Ar-H-5), 6.93 (d, 2H, *J* = 8.5 Hz, Ar-H-2, Ar-H-6), *6.90 (d, 2H, *J* = 8.5 Hz, Ar-H-2, Ar-H-6), 4.45 (q, 2H, *J* = 7.1 Hz, OC*H*_2_CH_3_), *4.36 (q, 2H, *J* = 7.1 Hz, OC*H*_2_CH_3_), 3.97-4.03 (m, 4H, OC*H*_2_CH_2_, *OC*H*_2_CH_2_), 1.76-1.84 (m, 4H, OCH_2_C*H*_2_, OCH_2_C*H*_2_*), 1.37-1.47 (m, 8H, 4×C*H*_2_), 1.33-1.37 (m, 6H, 2×C*H*_3_), 0.93 (t, 6H, *J* = 6.8 Hz, C*H*_3_, *C*H*_3_); ^13^C NMR (150 MHz, CDCl_3_): *δ* 162.9, 162.5, 161.8, 159.6, 138.0, 136.6, 133.0 (two), 132.7 (two), 132.2 (two), 121.1, 121.0, 115.4 (two), 115.3 (two), 68.4, 68.3, 62.9, 62.7, 28.7 (two), 28.1 (two), 22.4 (two), 14.1, 13.9 (two), 13.8;

HRESIMS calcd for C_16_H_21_NO_5_Na 330.3358; found 330.3359.

**(*Z* and *E*) Ethyl-3-(2-pentyloxyphenyl)-2-nitroacrylate (I-19)**

Yellow solid, yield 55%; ^1^H NMR (600 MHz, CDCl_3_): *δ* 8.50 (s, 1H, C=C*H*), *7.99 (s, 1H, C=C*H*), 7.38-7.47 (m, 3H, Ar-H), 7.32 (d, 1H, *J* = 7.8 Hz, Ar-H), 6.65-6.91 (m, 4H, Ar-H), 4.34-4.43 (m, 4H, 2×OC*H*_2_CH_3_), 4.00-4.07 (m, 4H, 2×OC*H*_2_CH_2_), 1.80- 1.88 (m, 4H, 2×OCH_2_C*H*_2_), 1.41-1.47 (m, 8H, 4×C*H*_2_), 1.36 (t, 3H, *J* = 7.1 Hz, C*H*_3_), *1.32 (t, 3H, *J* = 7.1 Hz, C*H*_3_), 0.95 (t, 6H, *J* = 7.0 Hz, 2×C*H*_3_) ; ^13^C NMR (150 MHz, CDCl_3_): *δ* 161.5, 159.6, 158.6, 158.0, 141.8, 140.2, 134.1, 133.6, 132.4, 129.5, 128.6, 128.5, 121.0, 120.6, 118.4 (two), 112.3, 112.1, 68.9, 68.8, 62.8 (two), 28.6, 28.5, 28.2 (two), 22.4 (two), 14.1 (two), 14.0 (two), 13.7; HRESIMS calcd for C_16_H_21_NO_5_Na 330.3358; found 330.3360.

**(*Z* and *E*) Ethyl-3-(4-acetoxyphenyl)-2-nitroacrylate (I-20)**

Yellow solid, yield 42%; ^1^H NMR (600 MHz, CDCl_3_): *δ* 8.05 (s, 1H, C=C*H*), 7.55 (d, 2H, *J* = 8.3 Hz, Ar-H-3, Ar-H-5), *7.51 (s, 1H, C=C*H*), *7.45 (d, 2H, *J* = 8.2 Hz, Ar-H-3, Ar-H-5), 7.21 (d, 2H, *J* = 8.3 Hz, Ar-H-2, Ar-H-6), *7.17 (d, 2H, *J* = 8.1 Hz, Ar-H-2, Ar-H-6), 4.44 (q, 2H, *J* = 7.1 Hz, OC*H*_2_CH_3_), *4.38 (q, *J* = 7.1 Hz, 2H, OC*H*_2_CH_3_), 2.32 (s, 3H, COC*H*_3_), *2.31 (s, 3H, COC*H*_3_), 1.34-1.39 (m, 6H, 2×C*H*_3_, *2×CH_3_); ^13^C NMR (150 MHz, CDCl_3_): *δ* 168.7 (two), 161.0, 159.1, 153.7, 153.4, 142.0, 140.1, 135.4 (two), 131.9, 131.7, 131.2 (two), 126.4, 126.3, 122.7 (two), 122.6 (two), 63.2, 63.1, 21.1 (two), 14.0, 13.7; HRESIMS calcd for C_13_H_13_NO_6_Na 302.2378; found 302.2381.

**(*Z* and *E*) Ethyl-3-(2-acetoxy-4-methoxyphenyl)-2-nitroacrylate (I-21)**

Yellow solid, yield 40%; ^1^H NMR (600 MHz, CDCl_3_): *δ* 8.00 (s, 1H, C=C*H*), *7.42 (s, 1H, C=C*H*), 7.33 (dd, 2H, *J* = 8.7, 2.2 Hz, Ar-H-6, Ar-H-6*), 7.26 (d, 1H, *J* = 2.3 Hz, Ar-H-2), *7.13 (d, 1H, *J* = 2.3 Hz, Ar-H-2), 7.01 (d, 1H, *J* = 8.6 Hz, Ar-H-5), *6.98 (d, 1H, *J* = 8.7 Hz, Ar-H-5), 4.44 (q, 2H, *J* = 7.1 Hz, OC*H*_2_CH_3_), *4.36 (q, 2H, *J* = 7.1 Hz, OC*H*_2_CH_3_), 3.90 (s, 1H, OC*H*_3_), *3.88 (s, 3H, OC*H*_3_), 2.32 (s, 3H, COC*H*_3_), *2.31 (s, 3H, COC*H*_3_), 1.35 (t, 6H, *J* = 7.1 Hz, CH_2_C*H*_3_, CH_2_C*H*_3_*); ^13^C NMR (150 MHz, CDCl_3_): *δ* 168.5 (two), 161.4, 159.4, 154.8, 154.6, 140.7, 140.2, 138.8, 135.7, 131.7, 130.9, 130.0, 128.9, 126.1, 124.9, 124.4, 121.5, 112.7, 112.6, 63.2, 63.0, 56.2, 56.1, 20.5 (two), 14.1, 13.7; HRESIMS calcd for C_14_H_15_NO_7_Na 332.2638; found 332.2640.

**(*Z* and *E*) Ethyl-3-(2-acetoxyphenyl)-2-nitroacrylate (I-22)**

Yellow solid, yield 38%; ^1^H NMR (600 MHz, CDCl_3_): *δ* 8.13 (s, 1H, C=C*H*), *7.65 (s, 1H, C=C*H*), 7.49-7.53 (m, 2H, Ar-H), 7.42 (dd, 1H, *J* = 7.9, 1.2 Hz, Ar-H), 7.25-7.31 (m, 2H, Ar-H), 7.24 (d, 1H, *J* = 8.2 Hz, Ar-H), 7.21 (dd, 1H, *J* = 8.2, 0.7 Hz, Ar-H), 4.38 (q, 4H, *J* = 7.1 Hz, OC*H*_2_CH_3_, *OC*H*_2_CH_3_), 2.37 (s, 3H, COC*H*_3_), *2.36 (s, 3H, COC*H*_3_), 1.37 (t, 3H, *J* = 7.1 Hz, CH_2_C*H*_3_), *1.28 (t, 3H, *J* = 7.2 Hz, CH_2_C*H*_3_); ^13^C NMR (150 MHz, CDCl_3_): *δ* 168.6 (two), 160.5, 158.9, 149.9, 149.4, 143.6, 141.9, 133.0, 132.8, 131.1, 129.4, 128.6, 127.7, 126.7, 126.3, 123.3, 123.2, 122.5, 122.3, 63.2, 63.1, 20.9, 20.8, 14.0, 13.7; HRESIMS calcd for C_13_H_13_NO_6_Na 302.2378; found 302.2381.

**(*Z* and *E*) Ethyl-3-(3,4-di-acetoxy-phenyl)-2-nitroacrylate (I-23)**

Yellow solid, yield 35%; ^1^H NMR (600 MHz, CDCl_3_): *δ* 8.01 (s, 1H, C=C*H*), *7.47 (s, 1H, C=C*H*), 7.40-7.43 (m, 1H, Ar-H), 7.32 (dd, 1H, *J* = 8.5, 2.2 Hz, Ar-H-6), 7.30 (d, 1H, *J* = 8.8 Hz, Ar-H), 7.27 (d, 2H, *J* = 2.3 Hz, Ar-H-2, *Ar-H-2), 4.43 (q, 2H, *J* = 7.2 Hz, OC*H*_2_CH_3_), *4.38 (q, 2H, *J* = 7.1 Hz, OC*H*_2_CH_3_), *2.31 (s, 3H, COC*H*_3_), 2.30 (s, 3H, COC*H*_3_), 1.36 (t, 6H, *J* = 7.1 Hz, CH_2_C*H*_3_, CH_2_C*H*_3_*); ^13^C NMR (150 MHz, CDCl_3_): *δ* 167.7 (two), 167.6, 167.5, 160.7, 158.9, 145.1, 145.0, 142.7, 142.6, 140.5, 134.6, 130.9 (two), 128.9, 128.2, 127.4, 127.3, 125.3, 124.9, 124.5, 124.4, 63.4, 63.3, 20.6 (two), 20.5 (two), 14.0, 13.7; HRESIMS calcd for C_15_H_15_NO_8_Na 360.2738; found 360.2740.

**(*Z* and *E*) 4-(3-ethoxy-2-nitro-3-oxoprop-1-en-1-yl)-2-methoxyphenyl benzoate (I-24)**

Yellow solid, yield 41%; ^1^H NMR (600 MHz, CDCl_3_): *δ* 8.16-8.26 (m, 4H, Ar-H), 8.08 (s, 1H, C=C*H*), 7.65 (t, 2H, *J* = 6.8 Hz, Ar-H), 7.50-7.55 (m, 5H, Ar-H, C=C*H* *), 7.25 (d, 1H, *J* = 8.4 Hz, Ar-H), 7.22 (d, 1H, *J* = 8.2 Hz, Ar-H), 7.20 (d, 2H, *J* = 7.4 Hz, Ar-H), 7.10 (d, 1H, *J* = 8.2 Hz, Ar-H), 7.05 (s, 1H, Ar-H), 4.44 (q, 2H, *J* = 7.1 Hz, OC*H*_2_CH_3_), *4.39 (q, *J* = 7.1 Hz, 2H, OC*H*_2_CH_3_), 3.83 (s, 3H, OC*H*_3_), *3.81 (s, 3H, OC*H*_3_), 1.37 (t, 6H, *J* = 7.1 Hz, 2×C*H*_3_); ^13^C NMR (150 MHz, CDCl_3_): *δ* 164.2, 164.2, 161.2, 159.2 , 152.0, 151.9, 143.4, 143.2, 142.1, 140.2, 135.9, 133.9, 133.8, 132.2, 130.4 (four), 128.9, 128.8, 128.7 (two), 128.6 (two), 127.7, 127.6, 124.0 (two), 123.9, 123.4, 113.8, 112. 9, 63.3, 63.2, 56.0 (two), 14.1, 13.8; HRESIMS calcd for C_19_H_17_NO_7_Na 394.3348; found 394.3350.


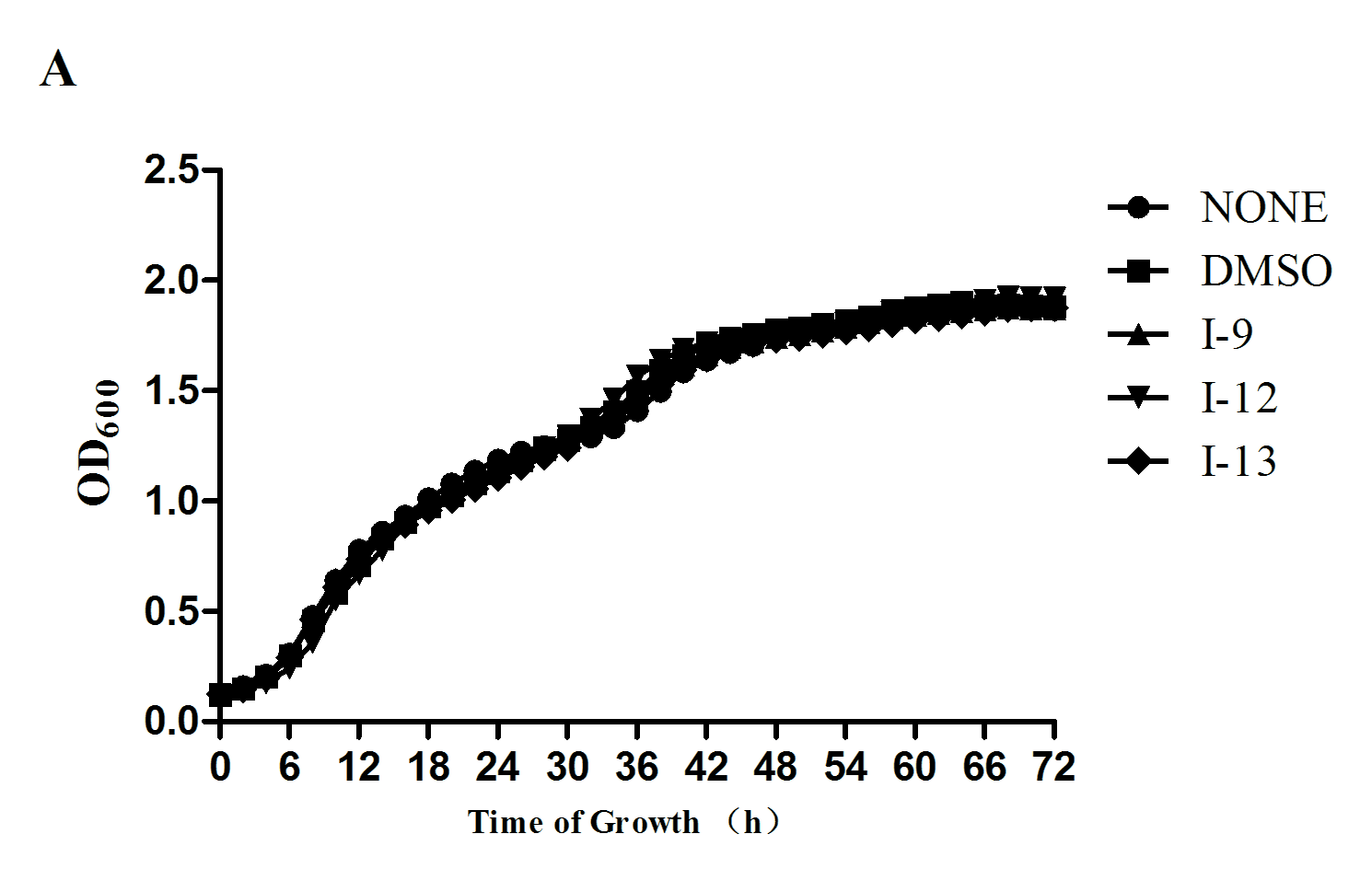

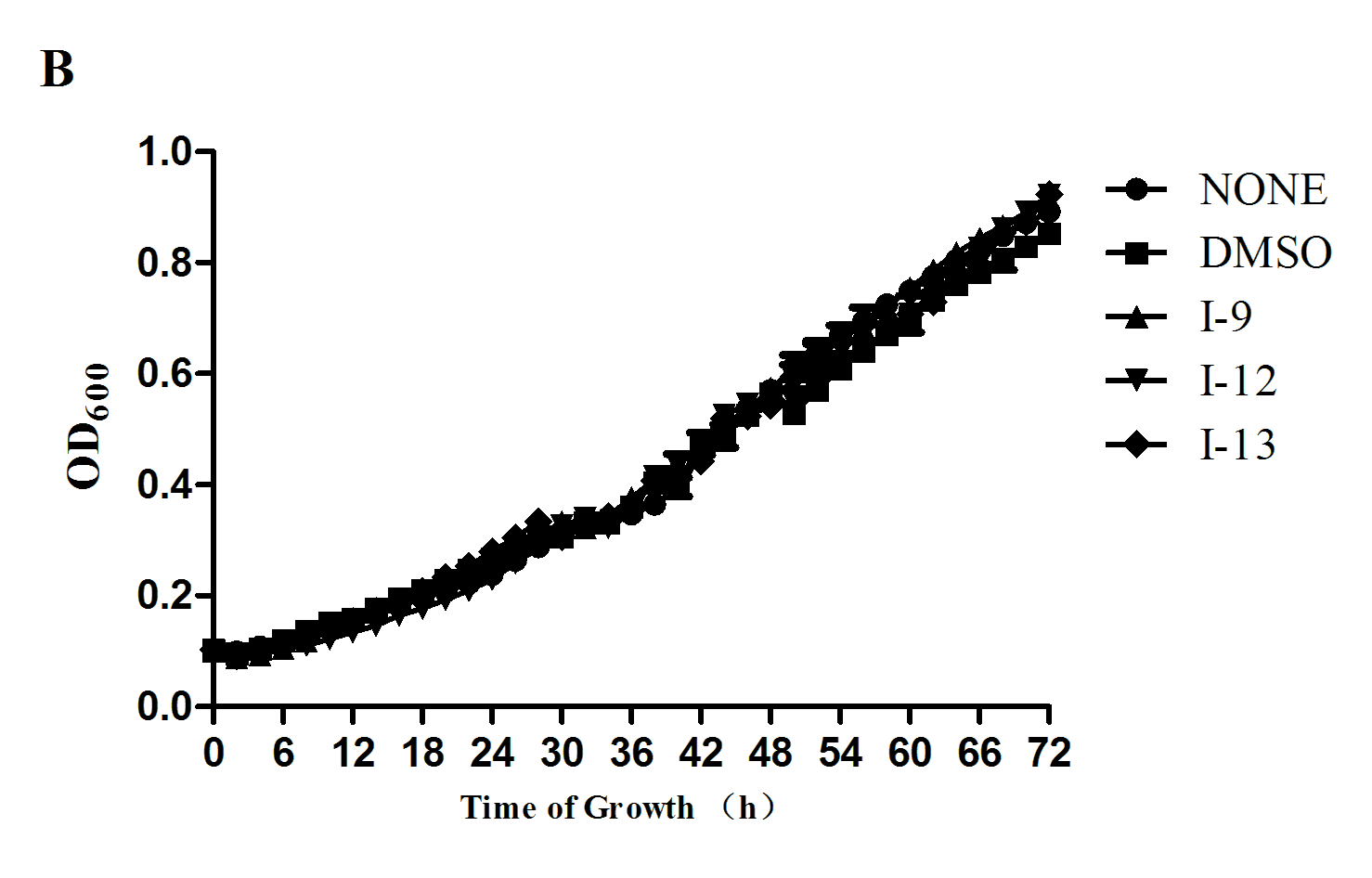


**Figure S5.** Effects of three compounds (I-9, I-12, and I-13) on bacterial growth rates. (A) The growth rate of *Xanthomonas oryzae* pv*. oryzae* (*Xoo*) PXO99^A^ in rich medium (M210) supplemented with DMSO (dimethylsulfoxide) or respectively 100 µM of I-9, I-12, and I-13. (B) The growth rate of *Xoo* PXO99^A^ in hrp-inducing medium (XOM2 plus 0.5% sucrose) supplemented with DMSO or 100 µM of I-9, I-12, and I-13 respectively. The optical density at 600 nm (OD_600_) of the culture suspensions was recorded every 2 h during the 72 h period.


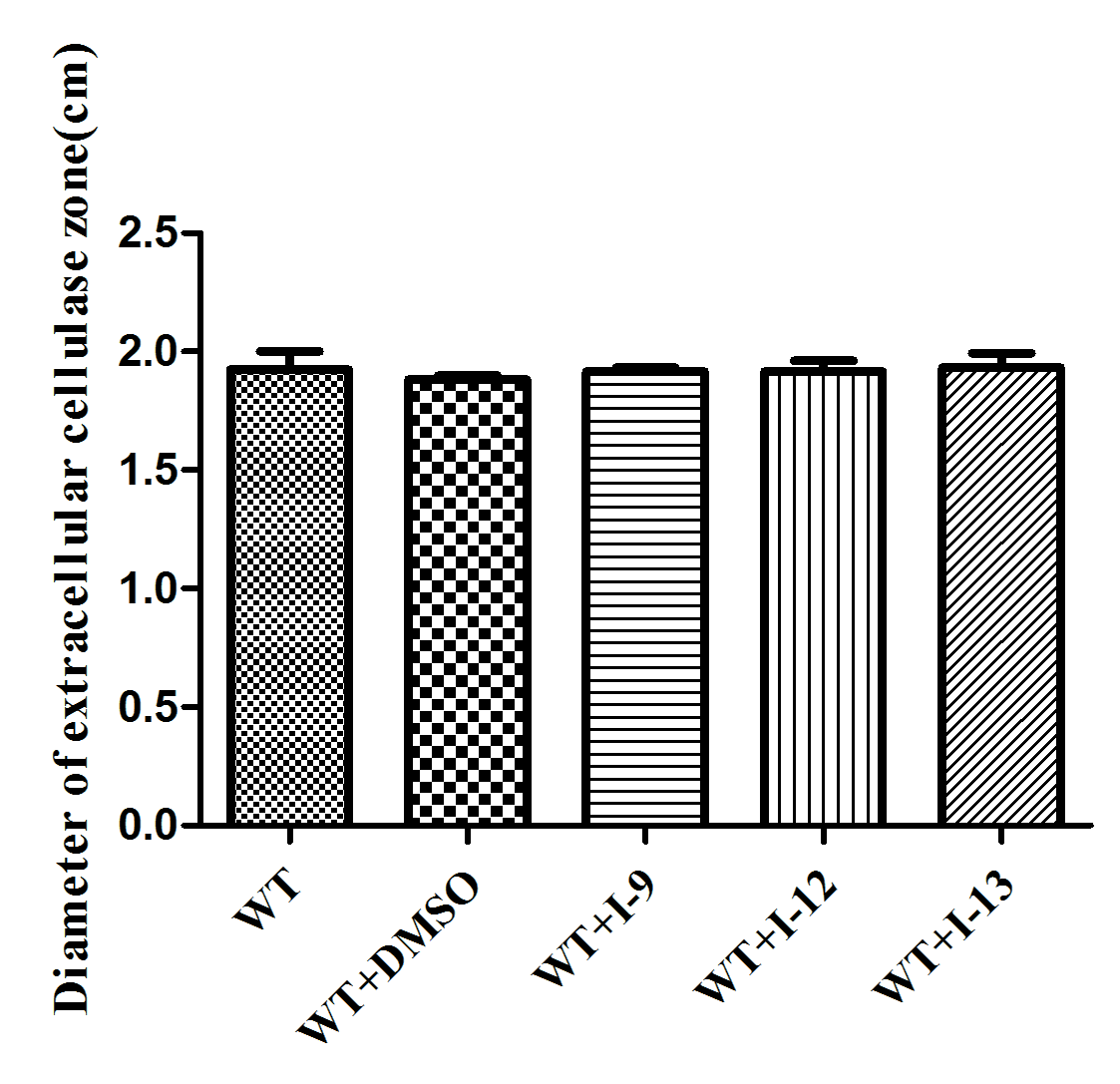


**Figure S6** The diameter of extracellular cellulase zone

**
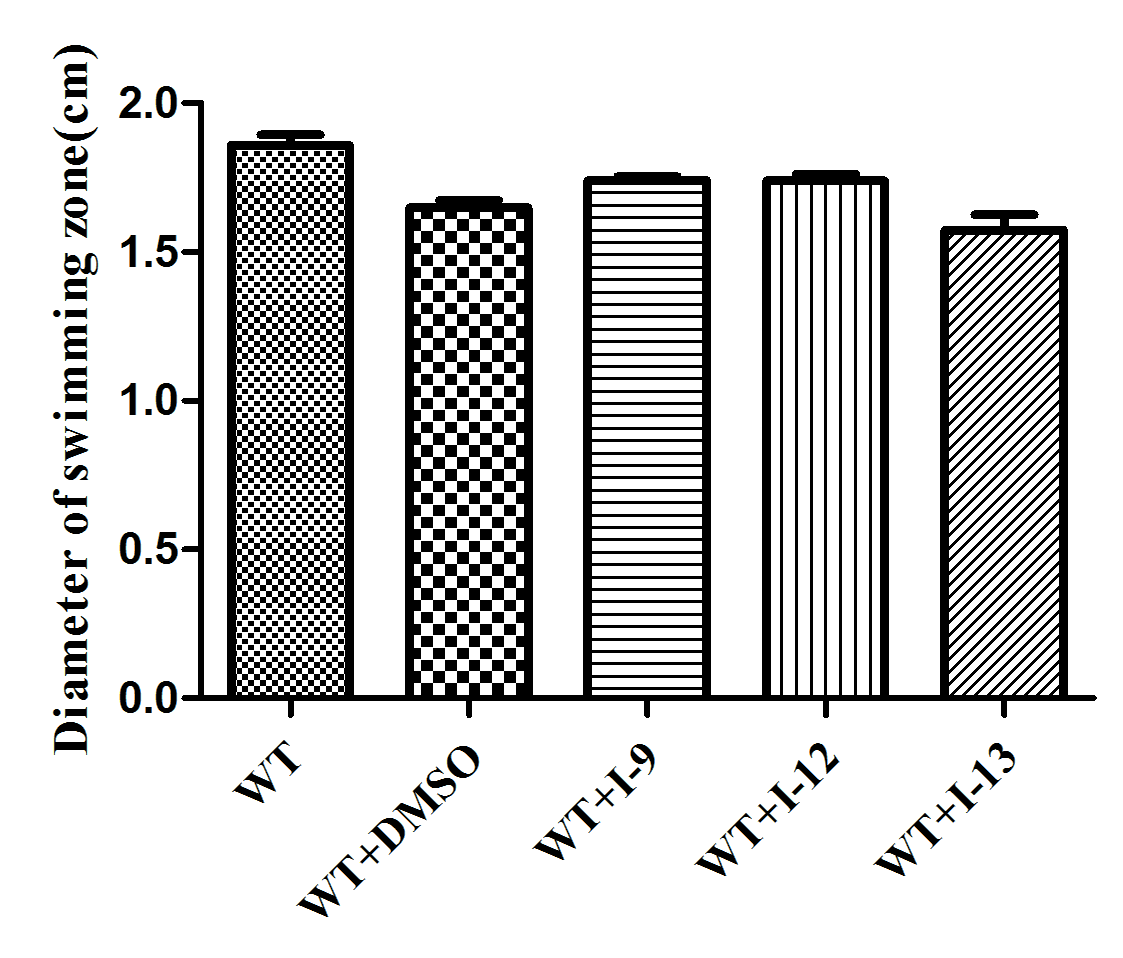
**

**Figure S7** The diameter of swimming zone


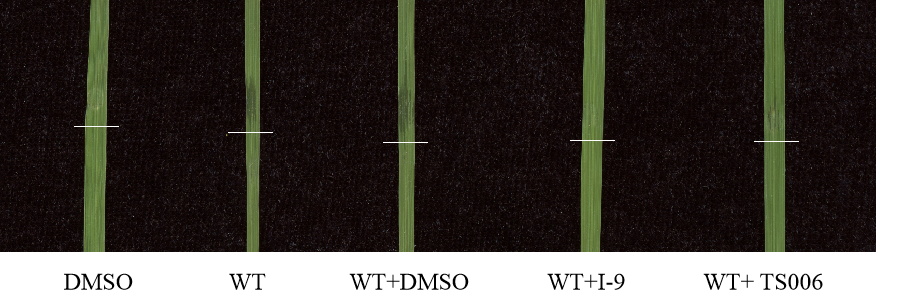


B

A


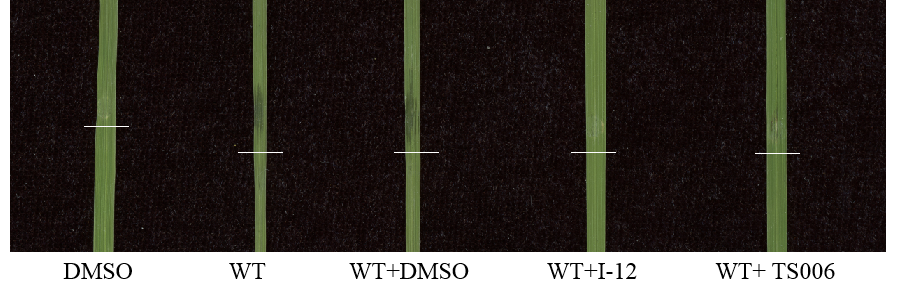


C


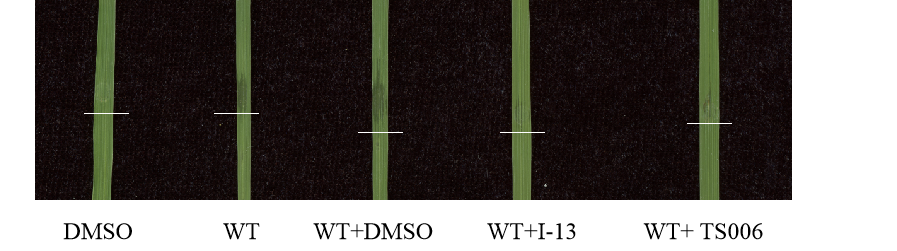


**Figure S8** (A, B, C) The effect of I-9, I-12, and I-13 on the water-soaking symptoms caused by *Xoo* wild-type on IR24 seedling.


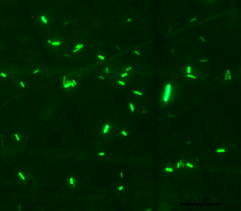

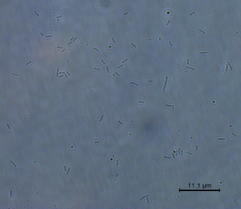

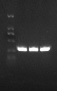

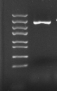


D

C

B

A

**Figure S9**. (A) The fluorescence spectrum of pPhpa1-*Xcc*8004. (B) The spectrum of pPhpa1-*Xcc*8004 under bright field conditions. (C) Amplification of *hpa1*fragments in *Xcc* by PCR. (D) PCR confiramtion of *Xcc*8004 carrying pPhpa1.
